# Pathology-targeted PET/CT imaging of early lupus nephritis via a ^68^Ga-labeled single-stranded DNA probe

**DOI:** 10.1126/sciadv.aei3589

**Published:** 2026-11-13

**Authors:** Zhiwen Huang, Wenbo Li, Meijiang Zhou, Zhidie Huang, Yingying Wei, Zhangyi Yin, Chenxi Yang, Zijun Ma, Min Shen, Quan Gong, Zachary Braunstein, Xiaoquan Rao, Dawei Jiang, Jixin Zhong

**Affiliations:** ^1^ Department of Rheumatology and Immunology, Fujian Medical University Union Hospital, Fuzhou, Fujian 350001, China.; ^2^ Department of Internal Medicine, Tongji Hospital, Tongji Medical College, Huazhong University of Science and Technology, Wuhan, Hubei 430030, China.; ^3^ Department of Nuclear Medicine, Union Hospital, Tongji Medical College, Huazhong University of Science and Technology, Wuhan, 430022, China.; ^4^ Department of Immunology, School of Medicine, Yangtze University, Jingzhou, Hubei 434023, China.; ^5^ Division of Hematology, Department of Internal Medicine, James Comprehensive Cancer Center, The Ohio State University Wexner Medical Center, Columbus, OH 43210, USA.; ^6^ Division of Cardiology, Department of Internal Medicine, Tongji Medical College and State Key Laboratory for Diagnosis and Treatment of Severe Zoonotic Infectious Diseases, Tongji Hospital, Huazhong University of Science and Technology, Wuhan, 430030, China.; ^7^ Hubei Key Laboratory of Molecular Imaging, Wuhan 430022, China.; ^8^ Key Laboratory of Biological Targeted Therapy (Huazhong University of Science and Technology), Ministry of Education, Wuhan, Hubei 430022, China.; ^9^ Key Laboratory of Vascular Aging (HUST), Ministry of Education, Wuhan, Hubei 430030, China.; ^10^ Fujian Institute of Clinical Immunology, Fuzhou, Fujian 350001, China.

## Abstract

As a major cause of morbidity in systemic lupus erythematosus (SLE), the prognosis of lupus nephritis (LN) critically depends on early detection and timely intervention. However, current diagnostic approaches remain suboptimal: renal biopsy is invasive and unsuitable for longitudinal monitoring, while conventional urine tests lack sensitivity for early-stage disease. Here, we report a molecular imaging strategy designed to target the pathogenic DNA–anti-dsDNA antibody axis for non-invasive detection of LN. We engineered a gallium-68-labeled single-stranded DNA (ssDNA) probe ([^68^Ga]Ga-NOTA-ssDNA) and evaluated its diagnostic performance and biosafety in the spontaneous MRL/lpr lupus mouse model. The probe showed disease-associated and sustained renal accumulation in 16-week-old diseased MRL/lpr mice, with progressively increasing kidney-to-background contrast. More importantly, [^68^Ga]Ga-NOTA-ssDNA enabled detection of renal immune complex deposition as early as 10 weeks of age, preceding abnormalities in conventional renal function markers and positive findings in renal histology by approximately two weeks. Mechanistically, the ssDNA backbone demonstrated favorable biocompatibility, without exacerbating autoimmune phenotypes or inducing Toll-like receptor-mediated signaling pathways. In conclusion, this study establishes a robust proof-of-concept for pathology-targeted PET/CT imaging of immune complex deposition. By directly visualizing the disease-driving renal autoantibody deposition at the preclinical stage, prior to overt organ damage, this strategy shifts LN diagnosis to non-invasive, mechanism-based molecular visualization.

## INTRODUCTION

Systemic lupus erythematosus (SLE) is a chronic, multi-system autoimmune disease characterized by immune complex deposition and episodic flares that result in cumulative organ damage (*1*, *2*). Among its clinical manifestations, lupus nephritis (LN) remains one of the most common and severe complications, affecting approximately 50% of patients and contributing substantially to morbidity and mortality. Despite advances in immunosuppressive therapy, 5–10% of LN patients progress to end-stage renal disease within 10 years after diagnosis, underscoring the critical importance of early detection and timely therapeutic intervention (*3*–*5*).

The pathogenesis of LN is driven by the deposition of immune complexes within the subendothelial and/or subepithelial regions of the glomeruli, particularly those composed of anti–double-stranded DNA (anti-dsDNA) antibodies and nucleic acids. These immune complexes trigger complement activation, inflammatory cell recruitment, and progressive structural damage (*6*–*8*). Importantly, these molecular pathological events occur long before the onset of clinical manifestations such as proteinuria, highlighting an essential window for early detection of renal immune complex deposition, at a stage when intervention can prevent irreversible injury (*9*, *10*). However, current diagnostic strategies for LN are poorly aligned with this biology. Renal biopsy, the clinical gold standard, provides detailed histopathological information (*11*, *12*) but is invasive, prone to sampling bias, and unsuitable for longitudinal monitoring (*13*, *14*). Other commonly used clinical surrogate markers, including serum anti-dsDNA antibody titers, complement C3/C4 levels, serum creatinine, proteinuria, and urine protein-to-creatinine ratio (UPCR), can indirectly reflect renal injury, but are either nonspecific to LN or become abnormal only after substantial pathological damage, making them unsuitable for early LN diagnosis (*15*, *16*).

Molecular imaging offers a promising alternative for non-invasive in vivo visualization of disease-relevant biological processes at an early stage (*17*). Positron emission tomography (PET) combined with computed tomography (CT), in particular, offers high sensitivity and quantitative whole-body imaging by using radiolabeled probes (*18*). While PET imaging has been well explored in inflammatory and oncologic contexts, its application in autoimmune diseases has long been ignored, with most efforts focused on nonspecific markers of inflammation or metabolism, rather than disease-driving molecular interactions. In LN, no imaging strategy to date has successfully targeted the deposition of pathogenic anti-dsDNA antibody complexes, a central event in disease initiation and progression (*19*, *20*).

Here, we propose a conceptually distinct approach: mechanism-based imaging of autoimmune pathology in LN through direct targeting of the DNA–anti-dsDNA antibody axis. Leveraging the intrinsic binding affinity of anti-dsDNA antibodies for nucleic acids (*21*), we engineered a gallium-68–labeled single-stranded DNA probe ([^68^Ga]Ga-NOTA-ssDNA) designed to selectively bind anti-dsDNA autoantibodies and their deposition in the kidney. Unlike conventional probes that detect secondary inflammatory responses, this strategy aims to visualize the primary molecular drivers of LN, thereby enabling earlier and more specific diagnosis.

We further addressed a critical barrier to the use of nucleic acid–based probes in autoimmune settings—namely, their potential to exacerbate immune activation. Double-stranded DNA is known to trigger Toll-like receptor (TLR) signaling and amplify lupus pathology; therefore, we employed a ssDNA backbone to minimize immunogenicity while preserving target recognition. Using the spontaneous MRL/lpr lupus mouse model, which closely recapitulates human SLE pathophysiology, we systematically evaluated the probe’s targeting specificity, pharmacokinetics, diagnostic performance across disease stages, and immunological safety. Our findings demonstrate that [^68^Ga]Ga-NOTA-ssDNA enables early, non-invasive detection of LN by directly visualizing renal immune complex deposition prior to the onset of functional impairment. This work establishes a proof-of-concept for pathology-targeted PET/CT imaging in autoimmune disease and highlights a broadly applicable strategy for translating disease mechanisms into clinically actionable imaging biomarkers.

## RESULTS

### The [^68^Ga]Ga-NOTA-ssDNA probe enables precise PET/CT diagnosis of LN

As one of the most severe and potentially fatal organ complications of SLE, LN still lacks effective methods for early diagnosis (*22*). Based on the targeting recognition property of anti-dsDNA antibodies toward DNA structures, we designed and constructed a [^68^Ga]Ga-NOTA-ssDNA PET/CT radionuclide imaging probe, in which ^68^Ga is conjugated to a random-sequence ssDNA via NOTA-A20 (Fig. 1A). To validate its potential as a diagnostic tool for LN, we first characterized the disease phenotype in 16-week-old female MRL/lpr mice, an established spontaneous lupus model (*23*). These mice exhibited a comprehensive systemic autoimmune profile, including significant weight gain (fig. S1, A and B), severe skin lesions (fig. S1, C and D), and marked hepatosplenomegaly (fig. S1, E to H). Plasma analysis showed a robust “lupus signature,” characterized by significantly elevated levels of anti-dsDNA antibodies, anti-nuclear antibodies (ANA), and anti-Smith antibodies, together with decreased complement C3 levels (Fig. 1, B and C, fig. S1, I to K). Biochemical analyses showed that UPCR as well as plasma creatinine, blood urea nitrogen, ALT, and AST levels were significantly elevated in MRL/lpr mice compared with the control group, indicating marked hepatic and renal dysfunction (fig. S1, L to P). We further collected kidney tissues from both groups, measured their weight, and calculated renal indices, which were increased in MRL/lpr mice, suggesting possible structural renal damage. To confirm the presence of LN, kidney tissues from both groups were subjected to H&E, PAS, Masson staining, and IgG immunofluorescence staining. The results showed that, compared with the control group, kidney tissues of 16-week-old MRL/lpr mice exhibited typical pathological features of glomerulonephritis (Fig. 1, D to F). H&E staining showed enlarged glomeruli and inflammatory cell infiltration in the renal interstitium. PAS staining showed mesangial cell proliferation and expansion of the mesangial matrix. Masson staining revealed mild collagen deposition in glomerular and tubulointerstitial regions, indicating that 16-week-old MRL/lpr mice were in the active stage of LN. IgG immunofluorescence staining showed prominent IgG immune complex deposition in the glomeruli of MRL/lpr mice (Fig. 1G). Histopathological scoring and analysis of mean IgG fluorescence intensity further confirmed these findings, with significantly higher values in MRL/lpr mice than in controls, indicating that 16-week-old MRL/lpr mice had developed pronounced LN with structural damage (Fig. 1H). Correlation analysis underscored the rationale for our probe design, as anti-dsDNA antibody titers showed the strongest correlation with both renal functional decline and pathological damage scores (Fig. 1I).

**Fig. 1.**
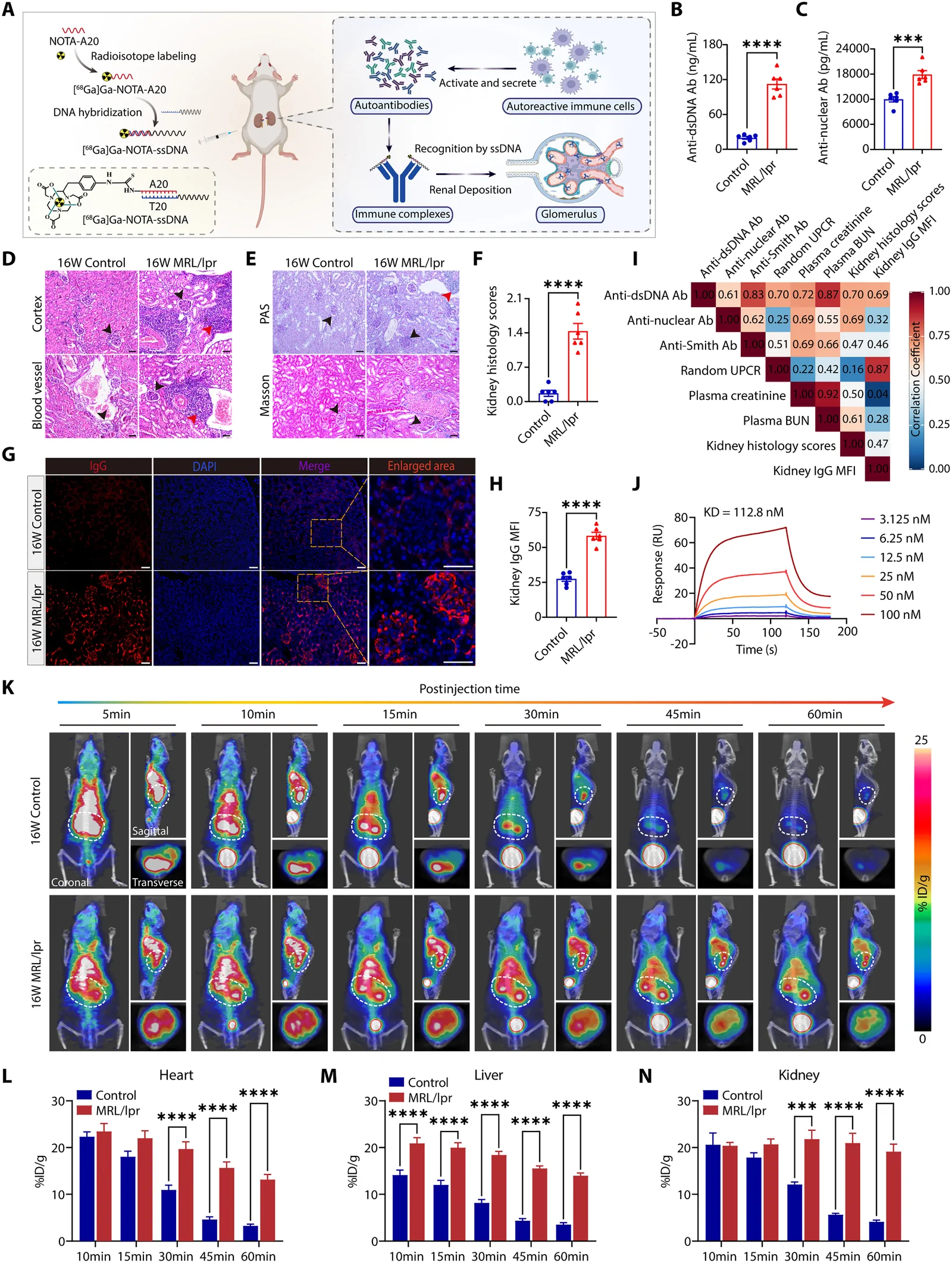
The [^68^Ga]Ga-NOTA-ssDNA probe enables precise PET/CT imaging diagnosis in MRL/lpr mice with LN. (**A**) Schematic illustration of the construction of the [^68^Ga]Ga-NOTA-ssDNA probe and its PET/CT imaging mechanism in MRL/lpr LN mice. (**B** and **C**) Comparison of plasma levels of anti-dsDNA antibody (B) and anti-nuclear antibody (C) between 16-week-old Control and MRL/lpr groups (*n* = 6). (**D** and **E**) Representative H&E (D), PAS and Masson (E) staining of kidney tissues from 16-week-old Control and MRL/lpr groups; black arrows indicate glomerular structures and red arrows indicate extensive lymphocyte infiltration; scale bar = 50 μm. (**F**) Comparison of renal histopathological scores between 16-week-old Control and MRL/lpr groups (*n* = 6). (**G**) Representative IgG immunofluorescence staining of kidney tissues from 16-week-old Control and MRL/lpr groups; scale bar = 50 μm. (**H**) Comparison of mean fluorescence intensity (MFI) of renal IgG deposition between 16-week-old Control and MRL/lpr groups (*n* = 6). (**I**) Correlation heatmap between SLE-specific autoantibodies and renal function biochemical indicators and renal immunopathology in 16-week-old MRL/lpr mice. (**J**) Binding affinity between the random-sequence ssDNA and anti-dsDNA antibodies assessed by surface plasmon resonance (SPR). (**K**) Maximum intensity projection (MIP) PET/CT images in coronal, sagittal, and transverse planes at 60 min after injection of [^68^Ga]Ga-NOTA-ssDNA probe in 16-week-old Control and MRL/lpr groups (kidney regions indicated by white dashed circles). (**L** to **N**) Quantitative ROI analysis of [^68^Ga]Ga-NOTA-ssDNA PET/CT imaging data in the heart (L), liver (M), and kidneys (N) of 16-week-old Control and MRL/lpr mice (*n* = 4). All data are presented as the mean ± SEM. Statistical analysis between two groups was performed using a two-tailed unpaired Student’s *t* test. Statistical comparisons among multiple groups were performed using two-way analysis of variance (Two-way ANOVA). ***P < 0.001, ****P < 0.0001.

We next evaluated the potential of the [^68^Ga]Ga-NOTA-ssDNA probe as a diagnostic tool for LN. Using surface plasmon resonance (SPR) affinity analysis, we first measured the binding affinity between the random-sequence ssDNA and anti-dsDNA antibodies, showing an equilibrium dissociation constant (KD) of 112.8 nM, indicating sufficient affinity for in vivo targeting consistent with the design expectations for targeting anti-dsDNA antibody probes (Fig. 1J). Subsequently, high-performance liquid chromatography (HPLC) was used to evaluate the structural stability of NOTA-A20 and [^68^Ga]Ga-NOTA-ssDNA at 0, 30, 60, and 120 minutes after synthesis, showing stability up to 60 minutes with noticeable dissociation at 120 minutes, meeting the requirements for subsequent 60-minute in vivo PET/CT imaging (fig. S2A). To evaluate the biosafety of the probe, [^68^Ga]Ga-NOTA-ssDNA was intravenously injected into mice, and major organs were collected for H&E histopathological analysis. The results showed no obvious histopathological damage in these organs at either time point compared with PBS controls, indicating good biocompatibility of the probe in mice (fig. S2B). After completing probe characterization, we conducted dynamic PET/CT imaging experiments in MRL/lpr mice. After tail vein injection of the probe, mice were immediately placed in a small-animal PET/CT scanner for 60-minute dynamic imaging. Coronal, sagittal, and transverse images showed that within 30 minutes postinjection, both MRL/lpr and control mice exhibited high circulating radiotracer uptake. From 30 to 60 minutes postinjection, the kidneys of MRL/lpr mice maintained high radiotracer uptake, whereas uptake in control mice rapidly declined, resulting in a clear imaging contrast between the two groups, indicating that the probe can distinguish LN mice from healthy controls and shows strong disease-associated renal retention in LN mice (Fig. 1K). We next quantitatively analyzed radiotracer uptake in regions of interest (ROIs) of the heart, liver, and kidneys based on dynamic PET/CT imaging data from 16-week-old MRL/lpr mice and age-matched controls. The results showed that significant differences in radiotracer uptake between the two groups emerged as early as 30 minutes postinjection, with markedly higher uptake in MRL/lpr mice than in controls, and the intergroup differences further increased over time (Fig. 1, L to N). At 60 minutes postinjection, the uptake values in the heart, liver, and kidneys of MRL/lpr mice were 13.17 ± 1.10% ID/g, 13.99 ± 0.57% ID/g, and 19.15 ± 1.59% ID/g, respectively, compared with 3.27 ± 0.36% ID/g, 3.53 ± 0.41% ID/g, and 4.14 ± 0.34% ID/g in the control group. The uptake in the heart and liver of MRL/lpr mice was approximately fourfold higher than that of controls, while renal uptake was about fivefold higher. To further verify the targeting specificity of the probe, we performed ex vivo autoradiography on kidney sections from both groups, which showed markedly stronger radioactive signals in the kidneys of 16-week-old MRL/lpr mice than in controls, providing tissue-level evidence consistent with preferential accumulation of [^68^Ga]Ga-NOTA-ssDNA in kidneys affected by lupus nephritis (fig. S2C).

### Pharmacokinetics and targeted biodistribution in mice with LN

To dynamically assess the distribution characteristics and pharmacokinetics of the [^68^Ga]Ga-NOTA-ssDNA probe in both groups, we plotted time-activity curves of probe uptake in the heart, liver, and kidneys. The results showed that in both the heart and liver, uptake curves in both groups exhibited an initial increase followed by a decrease. However, the decline in MRL/lpr mice was more gradual, and from 15 minutes postinjection onward, their curves remained consistently above those of the control group (Fig. 2, A and B). In the kidneys, the uptake curves differed markedly between groups: the control group showed a typical rise-and-fall pattern, whereas the MRL/lpr group exhibited a continuous increase over 60 minutes without an evident decline phase (Fig. 2C). Peak uptake time analysis showed no significant difference in cardiac time-to-peak between groups (approximately 1 minute). Hepatic time-to-peak was 4.38 ± 1.20 minutes in MRL/lpr mice versus 1.23 ± 0.27 minutes in controls. Renal time-to-peak was 32.5 ± 11.27 minutes in MRL/lpr mice versus 3 ± 0.29 minutes in controls, with all differences being statistically significant (Fig. 2, D to F). We then defined the 60-minute organ clearance rate as (peak uptake - uptake at 60 minutes)/peak uptake × 100% to quantitatively assess probe retention in each organ at 60 minutes postinjection. The results showed that the 60-minute clearance rates in MRL/lpr mice were 55.09 ± 2.78%, 35.61 ± 4.20%, and 18.49 ± 6.88% in the heart, liver, and kidneys, respectively, compared with 90.80 ± 1.00%, 82.70 ± 1.13%, and 84.89 ± 2.56% in controls. Clearance rates were significantly lower in all organs in MRL/lpr mice, with the kidneys showing the most pronounced difference, indicating marked probe retention likely associated with renal deposition of anti-dsDNA antibodies and immune complexes (Fig. 2, G to I).

**Fig. 2.**
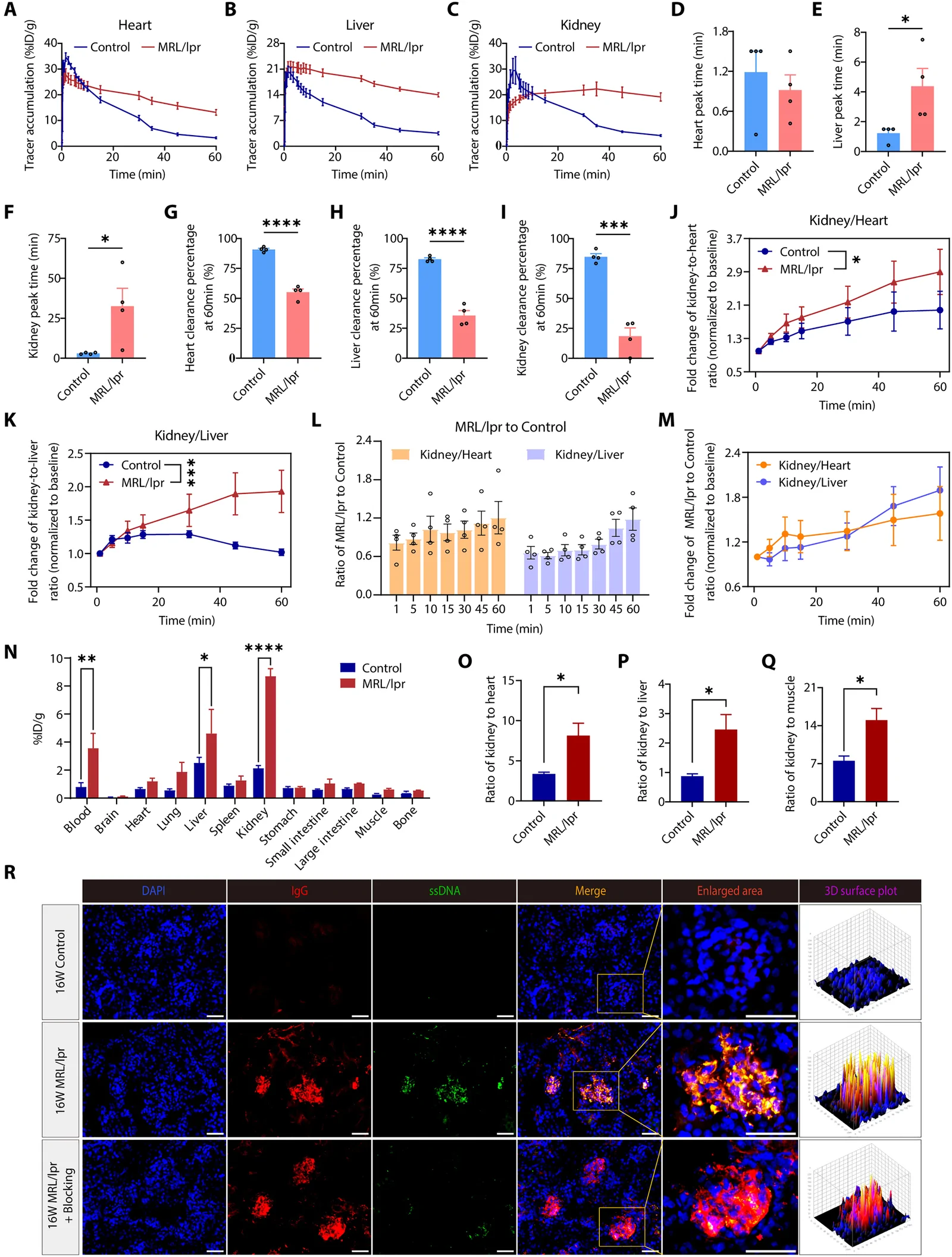
Pharmacokinetics and biodistribution of the [^68^Ga]Ga-NOTA-ssDNA probe in LN. (**A** to **C**) Time-activity curves of radiotracer uptake within 60 min after injection of the [^68^Ga]Ga-NOTA-ssDNA probe in the heart (A), liver (B), and kidneys (C) of 16-week-old Control and MRL/lpr mice (*n* = 4). (**D** to **F**) Comparison of time-to-peak of the [^68^Ga]Ga-NOTA-ssDNA probe in the heart (D), liver (E), and kidneys (F) between Control and MRL/lpr mice (*n* = 4). (**G** to **I**) Comparison of organ clearance rates at 60 min after injection of the [^68^Ga]Ga-NOTA-ssDNA probe in the heart (G), liver (H), and kidneys (I) (*n* = 4). (**J** and **K**) Time-dependent fold-change curves of kidney-to-heart (J) and kidney-to-liver (K) ratios within 60 min after injection of the [^68^Ga]Ga-NOTA-ssDNA probe (*n *= 4). (**L**) Quantification of kidney-to-heart and kidney-to-liver ratios at indicated time points after injection of [^68^Ga]Ga-NOTA-ssDNA between the two groups (*n* = 4). (**M**) Intergroup fold-change time curves of kidney-to-liver ratios within 60 min after injection of the [^68^Ga]Ga-NOTA-ssDNA probe between the two groups (*n* = 4). (**N**) Ex vivo biodistribution analysis of the [^68^Ga]Ga-NOTA-ssDNA probe in selected organs at 60 min postinjection (*n* = 4). (**O** to **Q**) Comparison of kidney-to-heart (O), kidney-to-liver (P), and kidney-to-muscle (Q) uptake ratios from ex vivo biodistribution at 60 min postinjection (*n* = 4). (**R**) Representative immunofluorescence images showing co-localization of IgG (red) and Cy5.5-labeled ssDNA probe (green) in kidney sections from Control, MRL/lpr, and MRL/lpr mice pre-injected with excess unlabeled ssDNA (competitive blockade); scale bar = 50 μm. All data are presented as the mean ± SEM. Statistical analysis between two groups was performed using a two-tailed unpaired Student’s *t* test. Statistical comparisons among multiple groups were performed using two-way analysis of variance (Two-way ANOVA). *P < 0.05, ***P < 0.001, ****P < 0.0001.

To assess the targeting specificity of the probe in LN, the kidney-to-heart and kidney-to-liver ratios at the start of imaging were normalized to the baseline, and the time-dependent fold changes in these ratios at subsequent imaging time points were plotted. The results showed that in the control group, these ratios fluctuated minimally throughout imaging, with no evident organ retention. In contrast, in the MRL/lpr group, the ratios gradually increased over time (Fig. 2, J and K), suggesting that the probe was specifically retained in the kidney tissue. This may be due to disease-related organ dysfunction in MRL/lpr mice, leading to slower systemic circulation and relatively lower peak uptake of the probe, resulting in lower kidney-to-heart and kidney-to-liver ratios in the early to mid imaging phases compared with controls. In the later imaging phase, due to the absence of probe retention in control mice and evident organ retention in MRL/lpr mice, the kidney-to-heart and kidney-to-liver ratios in MRL/lpr mice showed a continuous increase and eventually exceeded those in the control group. Further intergroup ratio curves based on kidney-to-heart and kidney-to-liver ratios at corresponding time points showed a progressive increase, indicating that with prolonged imaging time, radiotracer uptake in LN kidneys increased relative to background, while non-target organ signals from the heart and liver decreased, demonstrating good targeting specificity (Fig. 2, L and M).

To validate the in vivo PET/CT dynamic imaging results, ex vivo biodistribution studies were performed in both groups of mice at 60 minutes postinjection (Fig. 2N, table S1). The results showed that renal uptake in 16-week-old MRL/lpr mice was significantly higher than that in the control group (8.7008 ± 0.54 %ID/g vs. 2.1287 ± 0.1939 %ID/g, *P* < 0.0001). In addition, higher radiotracer uptake was observed in the blood and liver of MRL/lpr mice, with statistically significant differences between groups, consistent with in vivo imaging results. Ex vivo analysis further showed that kidney-to-heart, kidney-to-liver, and kidney-to-muscle ratios in 16-week-old MRL/lpr mice were all significantly higher than those in controls (8.1387 ± 1.5478 vs. 3.3744 ± 0.2048; 2.4573 ± 0.5117 vs. 0.8741 ± 0.0807; 15.0212 ± 2.1394 vs. 7.5352 ± 0.8794, respectively), with statistically significant differences (Fig. 2, O to Q). These results further demonstrate that the [^68^Ga]Ga-NOTA-ssDNA probe exhibits significantly enhanced renal radiotracer accumulation and prolonged circulation retention in LN mice, facilitating specific imaging of LN-affected kidneys.

To further assess the target specificity of the probe for renal immune complexes, we examined the deposition of the probe in renal tissue of 16-week-old mice in three groups: Control, MRL/lpr, and MRL/lpr subjected to competitive blockade via pre-injection of excess unlabelled ssDNA. All mice received a tail vein injection of Cy5.5-labelled ssDNA probe (Cy5.5-ssDNA, 0.5 nmol/g). Kidneys were harvested 60 min after injection and immunostained for IgG to assess its colocalization with Cy5.5-ssDNA. The kidneys of Control mice showed neither evident deposition of IgG immune complexes nor detectable Cy5.5-ssDNA signal, whereas MRL/lpr mice exhibited abundant glomerular IgG deposition with the majority co-localized with Cy5.5-ssDNA probe. Pretreatment with excess unlabelled ssDNA markedly abolished probe fluorescence while leaving IgG deposition essentially unchanged, indicating that probe accumulation at these sites depends on specific ssDNA binding (Fig. 2R). Collectively, these findings further demonstrate that the ssDNA probe specifically recognizes IgG-containing immune complexes, predominantly those containing anti-dsDNA antibodies, deposited in the kidneys of LN mice.

In summary, the [^68^Ga]Ga-NOTA-ssDNA probe showed strong renal targeting capability and diagnostic specificity in 16-week-old MRL/lpr mice with LN, supporting its potential application for PET/CT-based diagnosis of LN.

### Forward-shifting the diagnostic window of LN to the preclinical stage

To determine the earliest detectable imaging window, we performed longitudinal PET/CT imaging at four stages: 6 weeks (pre-disease), 10 weeks (preclinical stage), 12 weeks (early disease stage), and 16 weeks (mid-to-late stage), along with ex vivo biodistribution studies, and longitudinal evaluation of lupus-like phenotypes (Fig. 3A). We aimed to simulate the clinical progression of LN in SLE patients from absence to onset by selecting these four age time points in MRL/lpr mice, to evaluate the potential application and diagnostic window of the [^68^Ga]Ga-NOTA-ssDNA probe for early LN detection.

**Fig. 3.**
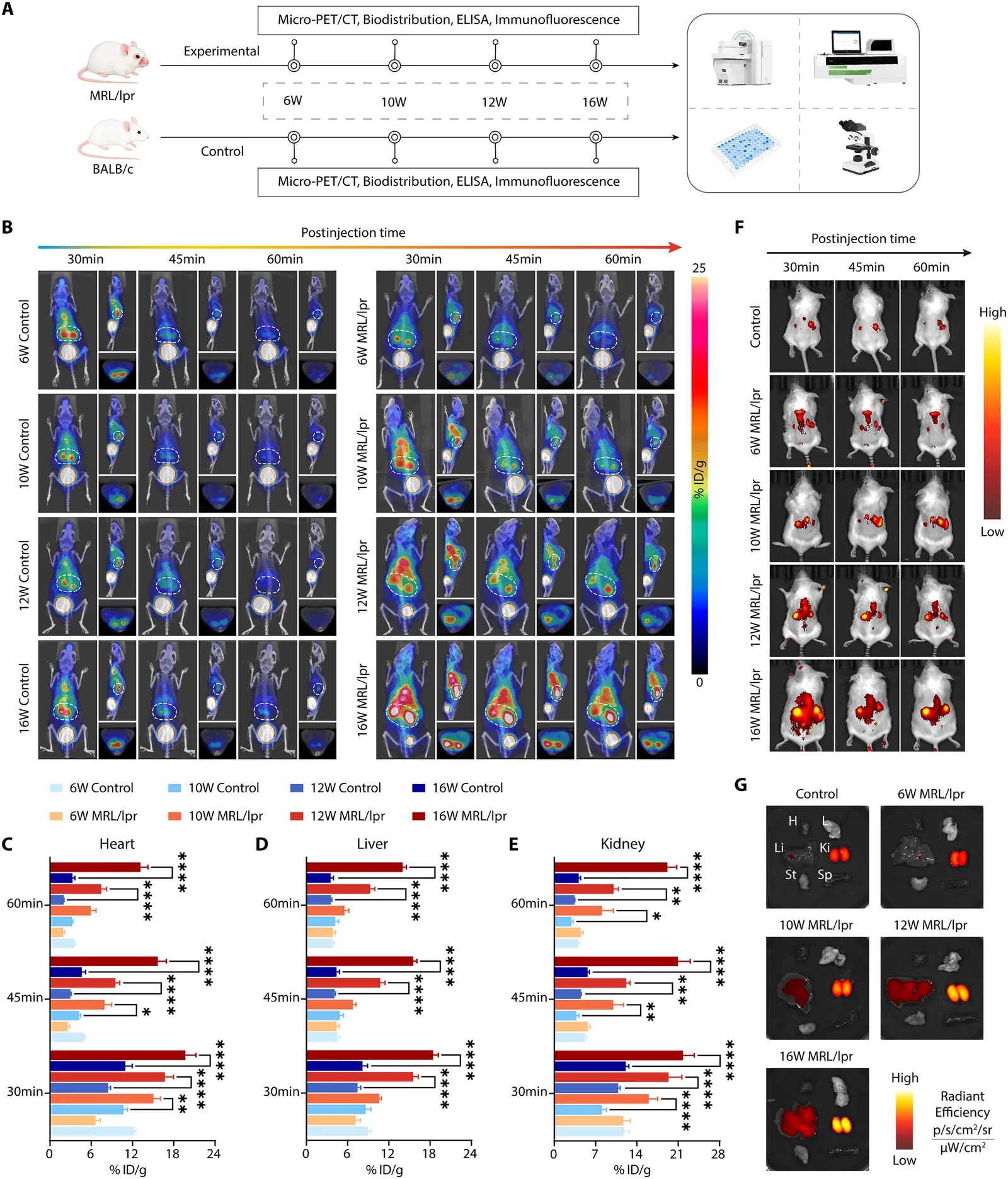
[^68^Ga]Ga-NOTA-ssDNA probe forward-shifts the diagnostic window of LN to the preclinical stage. (**A**) Schematic workflow of PET/CT imaging, biodistribution analysis, and lupus-like phenotype assessment using the [^68^Ga]Ga-NOTA-ssDNA probe in Control and MRL/lpr mice at 6, 10, 12, and 16 weeks of age. (**B**) Maximum intensity projection (MIP) PET/CT images in coronal, sagittal, and transverse planes at 60 min after injection of [^68^Ga]Ga-NOTA-ssDNA probe in Control and MRL/lpr mice at 6, 10, 12, and 16 weeks of age (kidney regions indicated by white dashed circles). (**C**) ROI-based quantitative analysis of cardiac PET/CT imaging data using the [^68^Ga]Ga-NOTA-ssDNA probe in Control and MRL/lpr mice at 6, 10, 12, and 16 weeks of age (*n* = 4). (**D**) ROI-based quantitative analysis of hepatic PET/CT imaging data using the [^68^Ga]Ga-NOTA-ssDNA probe in Control and MRL/lpr mice at 6, 10, 12, and 16 weeks of age (*n* = 4). (**E**) ROI-based quantitative analysis of renal PET/CT imaging data using the [^68^Ga]Ga-NOTA-ssDNA probe in Control and MRL/lpr mice at 6, 10, 12, and 16 weeks of age (*n* = 4). (**F**) Control and MRL/lpr mice of different ages underwent fluorescence imaging at 30, 45, and 60 min after Cy5.5-ssDNA injection (excitation/emission wavelengths, 754/778 nm). (**G**) Representative ex vivo fluorescence images of major organs. H, heart; L, lung; Li, liver; Ki, kidney; St, stomach; Sp, spleen. All data are presented as the mean ± SEM. Statistical comparisons among multiple groups were performed using two-way analysis of variance (Two-way ANOVA). *P < 0.05, **P < 0.01, ***P < 0.001, ****P < 0.0001.

Dynamic imaging results showed that control mice at all ages exhibited rapid metabolic clearance at approximately 30 minutes postinjection, with no evident renal radiotracer uptake at 45 and 60 minutes. The imaging profile of 6-week-old MRL/lpr mice was similar to that of age-matched controls, showing rapid clearance at 30 minutes postinjection and no significant difference in renal uptake. In 10-week-old MRL/lpr mice, PET/CT imaging differed markedly from controls, with sustained high renal uptake at 30 minutes postinjection; although clearance had begun, it was significantly slower than in controls, showing moderate renal retention and persistent imaging up to 60 minutes, possibly due to early deposition of anti-dsDNA antibodies in the kidneys. In 12-week-old MRL/lpr mice, renal imaging persisted up to 60 minutes, with consistently high tracer uptake throughout the imaging period, likely associated with increased production and organ deposition of anti-dsDNA antibodies. In 16-week-old MRL/lpr mice, the kidneys exhibited persistently high radiotracer uptake throughout imaging, with prominent focal accumulation, while the heart and liver also showed relatively elevated uptake, likely reflecting extensive production and widespread deposition of anti-dsDNA antibodies (Fig. 3B). The age-dependent enhancement of renal imaging was further confirmed by longitudinal monitoring of the same cohort of MRL/lpr mice at different ages, suggesting that this probe has considerable potential for the diagnosis and assessment of LN progression (fig. S3A).

To further quantitatively evaluate the organ uptake dynamics of the [^68^Ga]Ga-NOTA-ssDNA probe in mice of different ages, ROI-based analysis of the heart, liver, and kidneys was performed using PET/CT dynamic imaging data (Fig. 3, C to E). The results showed that at 6 weeks of age, there were no significant differences in uptake values in the heart, liver, or kidneys between MRL/lpr and control groups at any time point. At 10 weeks, renal uptake in the MRL/lpr group was significantly higher than that in the control group as early as 30 minutes postinjection, and the difference persisted to 60 minutes (8.05 ± 1.95 %ID/g vs. 2.87 ± 0.33 %ID/g, *P* < 0.05). From 12 weeks onward, radiotracer uptake in the heart, liver, and kidneys of MRL/lpr mice was significantly higher than that of age-matched controls at 30, 45, and 60 minutes postinjection, whereas controls maintained low uptake at all time points, with the magnitude of renal uptake changes being markedly greater than that in the heart and liver. Longitudinal analysis of age-dependent renal uptake showed that radiotracer accumulation in the kidneys of MRL/lpr mice increased significantly with age, with statistically significant differences observed at 12 weeks and older compared with 6 weeks, and inter-age differences progressively increasing with disease progression, consistent with the disease course of MRL/lpr mice.

To further validate the above findings, we performed fluorescence imaging in all groups of mice using a Cy5.5-labeled random-sequence ssDNA probe at 30, 45, and 60 min after injection (Fig. 3F). The fluorescence imaging results were consistent with the dynamic PET/CT findings. MRL/lpr mice exhibited significantly higher renal accumulation than control mice as early as 10 weeks of age, and the accumulation signal increased progressively with age. Ex vivo fluorescence imaging further confirmed that the probe exhibited pronounced specific accumulation in the kidneys of MRL/lpr mice, with an age-dependent increase in signal intensity (Fig. 3G). These findings further support the potential value of this probe for early LN detection at the tissue level.

### Pharmacokinetics and targeting specificity of the [^68^Ga]Ga-NOTA-ssDNA probe across different stages of LN

To further investigate the pharmacokinetic characteristics of the [^68^Ga]Ga-NOTA-ssDNA probe in MRL/lpr mice at different ages, we plotted time-activity curves of probe uptake in the heart, liver, and kidneys at 6, 10, 12, and 16 weeks (Fig. 4, A to C). The results showed that the time-activity curves of probe uptake in the heart, liver, and kidneys of control mice remained relatively consistent across ages, with no significant effect of age on organ uptake. In contrast, MRL/lpr mice exhibited a progressive elevation in the later phase of uptake curves in the heart, liver, and kidneys with increasing age, with the most pronounced changes observed in the kidneys, indicating a strong age-dependent pattern. Starting from 10 weeks of age, the later segments of the uptake curves in the heart, liver, and kidneys of MRL/lpr mice became markedly higher than those of age-matched controls, with the upward trend becoming more pronounced with increasing age. At 16 weeks, MRL/lpr mice exhibited pronounced probe retention in the kidneys with no clear clearance trend, consistent with the extensive production and renal deposition of anti-dsDNA antibodies during disease progression. Further analysis of renal time-to-peak uptake and 60-minute clearance rates showed that in MRL/lpr mice, peak time was progressively delayed and clearance rates decreased with age, with the greatest differences observed at 16 weeks compared with controls. Compared with age-matched controls, significant differences in renal time-to-peak were observed at 16 weeks, while differences in 60-minute renal clearance rates emerged as early as 12 weeks (Fig. 4, D and E). Analysis of peak time and 60-minute clearance in the heart and liver showed no significant differences in cardiac peak time between groups at any age, whereas hepatic peak time followed a pattern similar to renal peak time, with significant differences emerging only at 16 weeks. For 60-minute clearance rates in the heart and liver, significant differences between MRL/lpr and control groups were observed from 10 weeks onward, earlier than in the kidneys, possibly due to the probe first passing through cardiac and hepatic circulation before reaching the kidneys (fig. S3, B to E).

**Fig. 4.**
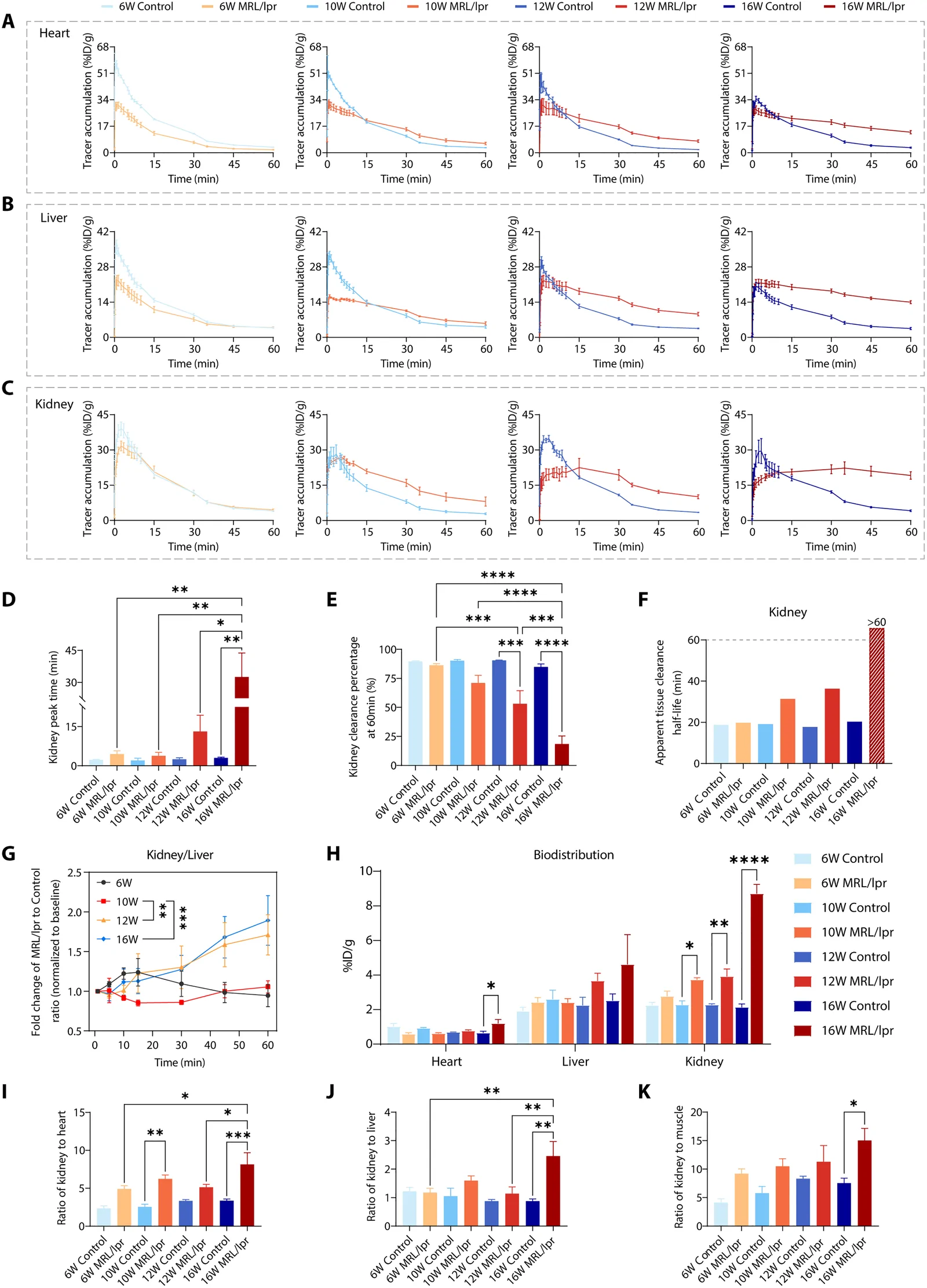
Pharmacokinetics, biodistribution, and targeting specificity of the [^68^Ga]Ga-NOTA-ssDNA probe across different stages of LN. (**A** to **C**) Time-activity curves of radiotracer uptake within 60 min after injection of the [^68^Ga]Ga-NOTA-ssDNA probe in the heart (A), liver (B), and kidneys (C) of Control and MRL/lpr mice at 6, 10, 12, and 16 weeks of age (*n* = 4). (**D**) Comparison of time to peak renal uptake of the [^68^Ga]Ga-NOTA-ssDNA probe in Control and MRL/lpr mice at 6, 10, 12, and 16 weeks of age (*n* = 4). (**E**) Comparison of renal clearance rates at 60 min postinjection of the [^68^Ga]Ga-NOTA-ssDNA probe in Control and MRL/lpr mice at 6, 10, 12, and 16 weeks of age (*n* = 4). (**F**) Comparison of renal apparent tissue clearance half-life (min) of the [^68^Ga]Ga-NOTA-ssDNA probe in Control and MRL/lpr mice at 6, 10, 12, and 16 weeks of age. (**G**) Intergroup fold-change time curves of kidney-to-liver ratios within 60 min after injection of the [^68^Ga]Ga-NOTA-ssDNA probe in MRL/lpr and Control mice at different ages (*n* = 4). (**H**) Ex vivo biodistribution analysis of the [^68^Ga]Ga-NOTA-ssDNA probe in selected organs at 60 min postinjection in Control and MRL/lpr mice of different ages (*n* = 4). (I to K) Comparison of kidney-to-heart (**I**), kidney-to-liver (**J**), and kidney-to-muscle (**K**) uptake ratios from ex vivo biodistribution at 60 min postinjection in Control and MRL/lpr mice across different ages (*n* = 4). All data are presented as the mean ± SEM. Statistical analysis for multiple-group comparisons was performed using one-way or two-way ANOVA as appropriate. *P < 0.05, **P < 0.01, ***P < 0.001, ****P < 0.0001.

Because conventional one- and two-compartment pharmacokinetic models are primarily designed to describe concentration changes in the central compartment (blood) following intravenous administration, they are not readily applicable to modeling the kinetics of probe uptake and clearance in renal tissue. To address this limitation, we integrated lupus-like phenotypic data from mice of different ages with a machine learning approach to establish a half-life estimation model that characterizes the uptake-clearance-retention (UCR) kinetics of [^68^Ga]Ga-NOTA-ssDNA in the kidneys. Comparison of model goodness-of-fit showed that the conventional decay-only model achieved an average R^2^ of only 0.38 when fitting renal time-activity curves, whereas the optimized UCR model increased the average R^2^ to greater than 0.95 (fig. S4A). Model-derived estimates showed that the apparent renal clearance half-life remained stable between 18 and 20 min across all ages in control mice. In contrast, the apparent renal clearance half-life in MRL/lpr mice was markedly prolonged from 10 weeks of age onward, exceeding 30 min (10 weeks: 31.39 min; 12 weeks: 36.33 min). At 16 weeks of age, the renal time-activity curve did not exhibit an obvious descending phase within the 60-min dynamic imaging window, preventing reliable estimation of the terminal clearance half-life; however, the apparent renal clearance half-life was determined to be substantially longer than 60 min (Fig. 4F). These findings further confirmed an age-dependent increase in the abnormal renal retention of [^68^Ga]Ga-NOTA-ssDNA in MRL/lpr mice, consistent with the progressive accumulation of immune complexes during LN progression. In addition, other pharmacokinetic parameters derived from the model, including C_max_ (the maximum renal radioactivity following probe injection), T_max_ (the time required to reach C_max_), AUC_0–60 min_ (the cumulative renal exposure to the radiotracer during the first 60 min after injection), and C60/C_max_ (the percentage of peak radioactivity remaining in the kidney at 60 min), exhibited age-dependent trends consistent with those of the apparent renal clearance half-life, further supporting these findings (fig. S4B).

Next, to further assess renal targeting of the [^68^Ga]Ga-NOTA-ssDNA probe across different ages in MRL/lpr mice, we calculated kidney-to-liver uptake ratios from PET/CT imaging data. The kidney-to-liver ratio curves in the control group showed no significant changes with age. In contrast, in MRL/lpr mice, the kidney-to-liver ratio curves showed a downward shift in the early phase and an upward shift in the late phase with increasing age, resulting in prolonged rising phases and disappearance of the declining phase (fig. S3F). Using the baseline kidney-to-liver ratio at imaging initiation, fold changes between MRL/lpr and age-matched control groups were calculated, showing a progressive upward trend with increasing age and significantly enlarged intergroup differences with disease progression (Fig. 4G).

To validate the PET/CT imaging results of the [^68^Ga]Ga-NOTA-ssDNA probe in MRL/lpr and control mice at different ages, ex vivo biodistribution studies were performed at 60 minutes postinjection (Fig. 4H and fig. S3G). The results showed that renal uptake in MRL/lpr mice was significantly higher than that in age-matched controls starting from 10 weeks, with intergroup differences increasing with age, and renal uptake in MRL/lpr mice showing an age-dependent increase, consistent with in vivo PET/CT imaging results; detailed biodistribution data are provided in Table 1. Furthermore, ex vivo biodistribution analysis showed that kidney-to-heart, kidney-to-liver, and kidney-to-muscle ratios between MRL/lpr and control groups increased progressively with age (Fig. 4, I to K).

**Table 1.** Biodistribution of [^68^Ga]Ga-NOTA-ssDNA across different ages*.

Tissue/organ	[^68^Ga]Ga-NOTA-ssDNA in 6 W Control	[^68^Ga]Ga-NOTA-ssDNA in 10 W Control	[^68^Ga]Ga-NOTA-ssDNA in 12 W Control	[^68^Ga]Ga-NOTA-ssDNA in 16 W Control	[^68^Ga]Ga-NOTA-ssDNA in 6 W MRL/lpr	[^68^Ga]Ga-NOTA-ssDNA in 10 W MRL/lpr	[^68^Ga]Ga-NOTA-ssDNA in 12 W MRL/lpr	[^68^Ga]Ga-NOTA-ssDNA in 16 W MRL/lpr
Blood (%ID/g)	0.9697 ± 0.1465	0.7112 ± 0.1611	0.5764 ± 0.0732	0.7970 ± 0.2978	0.2024 ± 0.0381	1.1049 ± 0.3481	2.1612 ± 0.7839	3.5616 ± 1.0557
Brain (%ID/g)	0.0586 ± 0.0057	0.0444 ± 0.0058	0.0603 ± 0.0098	0.0505 ± 0.0177	0.0273 ± 0.0013	0.0602 ± 0.0073	0.0859 ± 0.0165	0.1164 ± 0.0294
Heart (%ID/g)	1.0152 ± 0.1775	0.9171 ± 0.0512	0.6838 ± 0.0215	0.6480 ± 0.0988	0.5816 ± 0.0832	0.6092 ± 0.0560	0.7630 ± 0.0670	1.1918 ± 0.2322
Lung (%ID/g)	0.8823 ± 0.1689	0.7059 ± 0.1049	0.7585 ± 0.0763	0.5548 ± 0.1067	0.4024 ± 0.0759	0.6388 ± 0.0626	1.1044 ± 0.1824	1.8741 ± 0.6840
Liver (%ID/g)	1.9025 ± 0.2302	2.5906 ± 0.5359	2.6188 ± 0.1877	2.5132 ± 0.3872	2.4214 ± 0.2747	2.4051 ± 0.2254	3.6609 ± 0.4391	4.6096 ± 1.7227
Spleen (%ID/g)	0.9928 ± 0.0607	0.8042 ± 0.0171	1.0314 ± 0.0790	0.8852 ± 0.1212	0.6804 ± 0.1034	0.6902 ± 0.0567	0.9549 ± 0.0601	1.2661 ± 0.3022
Kidney (%ID/g)	2.2466 ± 0.1729	2.2794 ± 0.2269	2.2645 ± 0.0754	2.1287 ± 0.1939	2.7711 ± 0.3010	3.7224 ± 0.1164	3.9142 ± 0.4345	8.7008 ± 0.5400
Stomach (%ID/g)	1.4858 ± 0.4095	0.9428 ± 0.1138	0.7774 ± 0.1483	0.7185 ± 0.1061	0.5867 ± 0.0850	0.5300 ± 0.0645	0.5448 ± 0.0333	0.7489 ± 0.0725
Small intestine (%ID/g)	0.9988 ± 0.0522	0.6760 ± 0.0625	0.7729 ± 0.0458	0.5931 ± 0.0529	0.4924 ± 0.0732	0.6056 ± 0.0889	0.6606 ± 0.0474	1.0442 ± 0.3105
Large intestine (%ID/g)	1.4878 ± 0.3824	1.1309 ± 0.1298	0.9046 ± 0.0617	0.6497 ± 0.0746	0.7297 ± 0.1412	0.6097 ± 0.1119	0.7118 ± 0.0212	1.0415 ± 0.0261
Muscle (%ID/g)	0.6182 ± 0.1448	0.4523 ± 0.0928	0.2750 ± 0.0181	0.2941 ± 0.0391	0.3145 ± 0.0551	0.3765 ± 0.0580	0.4232 ± 0.1130	0.6094 ± 0.0762
Bone (%ID/g)	1.3233 ± 0.0543	0.9269 ± 0.0964	0.8619 ± 0.0908	0.3964 ± 0.0899	0.7551 ± 0.0467	0.7366 ± 0.0501	0.5699 ± 0.0270	0.5369 ± 0.0286

* Data are presented as the mean ± SEM (*n* = 4).

Collectively, these results indicate that the renal targeting specificity of the [^68^Ga]Ga-NOTA-ssDNA probe in MRL/lpr mice increases with the progression of LN, with a PET/CT diagnostic window as early as 10 weeks (approximately corresponding to the preclinical stage), suggesting its potential for early diagnosis of LN.

### Correlation between the PET/CT imaging characteristics and LN phenotypes

To investigate imaging differences of the [^68^Ga]Ga-NOTA-ssDNA probe across MRL/lpr mice of different ages and their intrinsic correlation with LN phenotypes, we first performed a systematic evaluation of lupus-like phenotypes in all groups. By plotting body weight changes relative to the 6-week baseline, we found that, except at 12 weeks, there were no significant differences in overall weight gain between the two groups. The significant increase in body weight in MRL/lpr mice at 12 weeks may be associated with disease onset, during which abnormal immune activation leads to hepatosplenomegaly and lymphadenopathy (Fig. 5A). Analysis of liver and spleen indices further confirmed that MRL/lpr mice exhibited mild hepatomegaly from 10 weeks, with significant increases in both indices at 12 weeks, indicating pronounced hepatosplenomegaly that progressively worsened with age (Fig. 5, B and C). We then measured SLE-specific autoantibody levels in plasma by ELISA, which showed that anti-dsDNA antibody levels in MRL/lpr mice began to increase from 10 weeks, showing significant differences compared with controls, whereas anti-nuclear and anti-Smith antibodies had not yet increased significantly at this time point (Fig. 5, D to F). These findings are consistent with the observation that the [^68^Ga]Ga-NOTA-ssDNA probe can already produce differential imaging in 10-week-old MRL/lpr mice compared with controls, further supporting the specific targeting interaction between the probe and anti-dsDNA antibodies. At 12 and 16 weeks, all three SLE-specific autoantibodies were abundantly produced in MRL/lpr mice, promoting the development of lupus-like phenotypes and driving LN-associated PET/CT imaging changes of the probe in these mice.

**Fig. 5.**
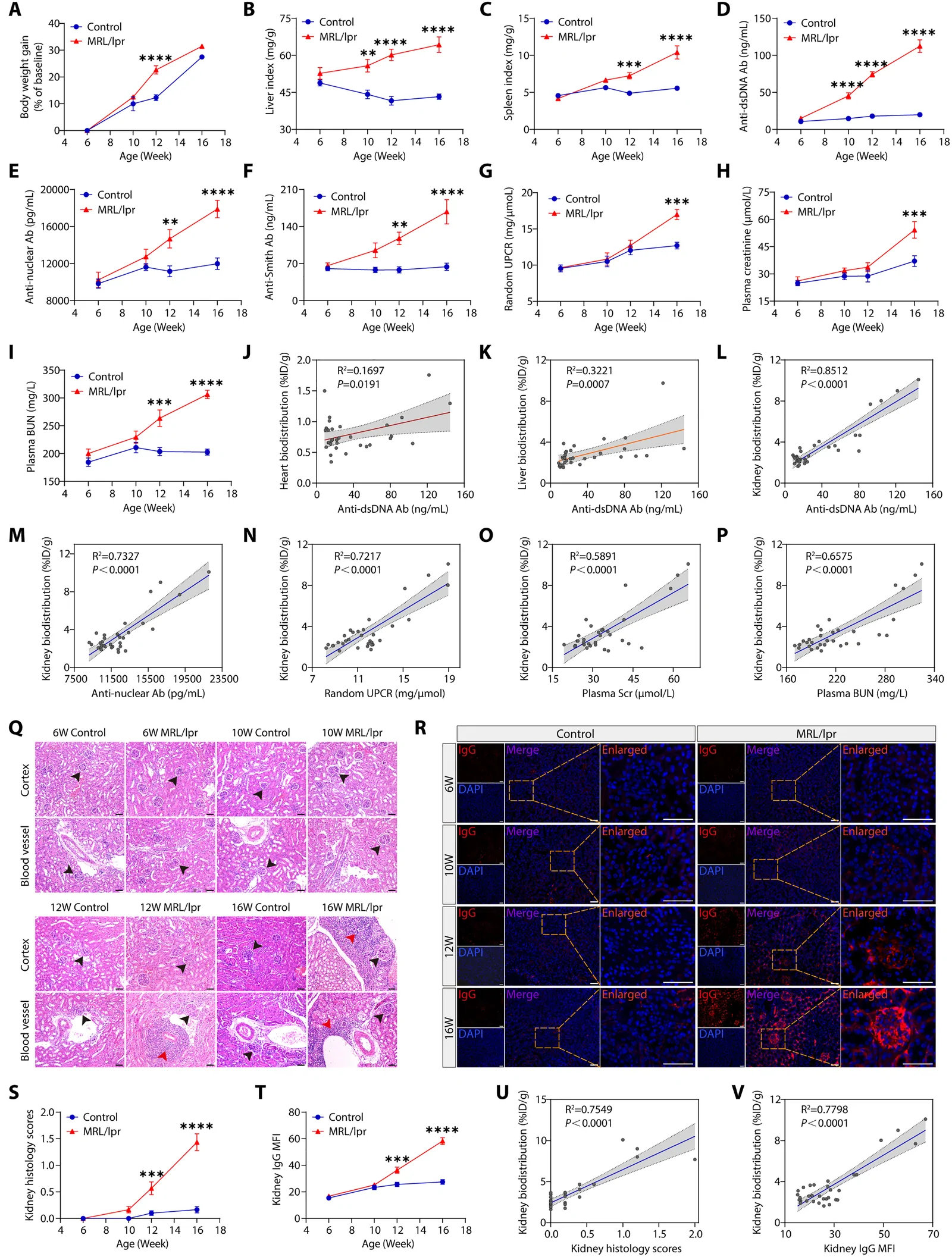
Correlation between the PET/CT imaging characteristics and lupus-like phenotypes in MRL/lpr mice at different ages. (**A**) Body weight gain curves relative to the 6-week baseline in Control and MRL/lpr mice at 6, 10, 12, and 16 weeks of age (*n* = 6). (**B** to **I**) Liver index (B), spleen index (C), plasma anti-dsDNA (D), anti-nuclear (E), anti-Smith (F) antibody, random UPCR (G), plasma creatinine (H), and blood urea nitrogen (I) level curves in Control and MRL/lpr mice at 6, 10, 12, and 16 weeks of age (*n* = 6). (**J** to **L**) Correlation analyses of cardiac (J), hepatic (K), and renal (L) tracer biodistribution with plasma anti-dsDNA antibody levels in Control and MRL/lpr mice. (**M** to **P**) Correlation analyses of renal tracer biodistribution with anti-nuclear antibody levels (M), random UPCR (N), plasma creatinine (O), and blood urea nitrogen (P) in Control and MRL/lpr mice. (**Q** and **R**) Representative H&E-stained (Q) and IgG immunofluorescence (R) images of kidneys from Control and MRL/lpr mice at different ages, with black arrows indicating glomerular structures and red arrows indicating extensive lymphocyte infiltration; scale bar = 50 μm. (**S** and **T**) Renal histopathological score curves (S) and mean fluorescence intensity (MFI) curves of renal IgG deposition (T) in Control and MRL/lpr mice across different ages (*n* = 6). (**U** and **V**) Correlation analyses of renal tracer biodistribution with renal histopathological scores (U) and mean fluorescence intensity (MFI) of renal IgG deposition (V) in Control and MRL/lpr mice. All data are presented as the mean ± SEM. Statistical analysis for multiple-group comparisons was performed using two-way ANOVA. Correlation analysis was performed using simple linear regression to calculate the correlation coefficient (R), and the corresponding 95% confidence interval (CI) was reported. **P < 0.01, ***P < 0.001, ****P < 0.0001.

To compare the diagnostic performance and detection window of the [^68^Ga]Ga-NOTA-ssDNA probe with conventional renal function indicators in LN, we measured the random UPCR, plasma creatinine, and blood urea nitrogen levels in all groups. The results showed that blood urea nitrogen levels in MRL/lpr mice began to increase significantly at 12 weeks, whereas the UPCR and plasma creatinine levels showed significant elevation only at 16 weeks (Fig. 5, G to I). These findings indicate that the earliest diagnostic window of conventional renal function indicators for LN in MRL/lpr mice is approximately 12 weeks, which is substantially later than the 10-week diagnostic window of probe imaging, suggesting a distinct advantage of this probe for the early detection of LN.

To further clarify the potential relationship between the PET/CT imaging performance of [^68^Ga]Ga-NOTA-ssDNA and the lupus nephritis phenotype in MRL/lpr mice, we performed correlation analyses between probe biodistribution in the heart, liver, and kidneys and the phenotypic data obtained from all experimental groups. The results showed that renal probe biodistribution was strongly and positively correlated with serum anti-dsDNA antibody levels (R^2^ = 0.8512), with a substantially stronger correlation than that observed for the heart (R^2^ = 0.1697) or liver (R^2^ = 0.3221), further supporting the renal targeting specificity of the probe in LN. In addition, renal probe biodistribution was well correlated with antinuclear antibody levels (R^2^ = 0.7327), UPCR (R^2^ = 0.7217), plasma creatinine (R^2^ = 0.5891), and blood urea nitrogen (R^2^ = 0.6575), indicating that renal probe accumulation is closely associated with multiple dimensions of the lupus-like phenotype and further supporting the reliability of the probe for the early diagnosis of LN.

We next performed hematoxylin and eosin (H&E) staining and IgG immunofluorescence staining on kidney sections from all groups to evaluate histopathological changes and immune complex deposition (Fig. 5, Q and R). H&E staining showed that at 6 and 10 weeks, no obvious glomerular morphological changes or interstitial inflammatory cell infiltration were observed in either MRL/lpr or control mice. At 12 weeks, MRL/lpr mice exhibited mild glomerular enlargement and perivascular inflammatory cell infiltration in the renal interstitium. By 16 weeks, renal tissues in MRL/lpr mice displayed typical pathological features of LN, including marked glomerular enlargement and extensive interstitial inflammatory infiltration. Histopathological scoring further confirmed that renal pathology scores in MRL/lpr mice increased progressively with age, showing significant differences from controls starting at 12 weeks and more pronounced differences at 16 weeks, indicating progressive structural damage (fig. S5). IgG immunofluorescence staining showed no evident IgG-positive signals in control mice at all ages or in 6- and 10-week-old MRL/lpr mice. At 12 weeks, IgG-positive fluorescence began to appear in MRL/lpr mice. By 16 weeks, pronounced IgG-positive deposition was observed. Quantitative analysis of mean fluorescence intensity further confirmed that IgG fluorescence in MRL/lpr kidneys increased progressively with age and showed significant differences from controls starting at 12 weeks (Fig. 5T). These findings are consistent with the histopathological scoring results and align with the age-dependent progression of LN in MRL/lpr mice. Correlation analysis showed that both the renal histopathological score (R^2^ = 0.7549) and the mean fluorescence intensity of IgG deposition (R^2^ = 0.7798) were strongly correlated with the renal biodistribution of the probe (Fig. 5, U and V), indicating that the extent of renal tissue injury and the burden of immune complex deposition are closely associated with the in vivo distribution of the probe in LN, further supporting its targeting specificity and disease relevance.

In summary, renal functional impairment and pathological features associated with LN in MRL/lpr mice begin to emerge around 12 weeks of age and can only be clearly identified by conventional diagnostic methods at 16 weeks. In contrast, the [^68^Ga]Ga-NOTA-ssDNA probe is able to generate differential PET/CT imaging in 10-week-old MRL/lpr mice compared with controls, at which stage anti-dsDNA antibodies have just begun to emerge, and no overt structural renal pathology is present. These findings indicate that the constructed [^68^Ga]Ga-NOTA-ssDNA probe showed early diagnostic potential for LN in this model, enabling a shift of the diagnostic window to the preclinical stage, detecting disease-associated changes earlier than conventional renal function tests and histopathological examination in this model, and offering potential for early imaging-based identification of at-risk patients.

### ssDNA probe demonstrates favorable safety for PET/CT imaging in LN

Anti-dsDNA antibodies are key pathogenic autoantibodies in LN, produced by autoreactive B cells upon exposure to large amounts of undegraded DNA, and they can form pathogenic immune complexes by recognizing various DNA structures and deposit in multiple organs throughout the body (*24*). Given the critical role of the DNA–anti-dsDNA antibody axis in LN pathogenesis, we evaluated the effect of long-term administration of ssDNA and dsDNA on LN progression. We administered PBS, ssDNA, or dsDNA (3 nmol per mouse) weekly for 5 weeks starting at 8 weeks of age (Fig. 6A). Body weight and UPCR were monitored weekly throughout the study. Body weight monitoring showed no significant differences between the ssDNA or dsDNA groups and the PBS group during the 5-week injection period. At 13–14 weeks, both ssDNA and dsDNA groups exhibited a transient but significant decrease in body weight, which recovered by 15 weeks to levels comparable to the PBS group, likely due to transient organ dysfunction caused by cumulative immunostimulation, with dsDNA showing a stronger effect than ssDNA (Fig. 6B). UPCR measurements supported this observation, showing increased UPCR at week 13 and 14 in the dsDNA group and slight transient increase in the ssDNA group at week 13 (Fig. 6C). Organ index analysis showed that liver, spleen, lymph node, and kidney indices were significantly increased in the dsDNA group compared with PBS, whereas the ssDNA group showed only slight, non-significant increases, indicating that ssDNA has substantially lower immunostimulatory effects than dsDNA (Fig. 6, D to G). ELISA analysis showed that compared with PBS controls, the dsDNA group exhibited significantly increased levels of anti-dsDNA, anti-nuclear, and anti-Smith antibodies, along with a significant decrease in complement C3 levels. In contrast, the ssDNA group showed mild increases in anti-dsDNA and anti-Smith antibodies only, with no significant differences in anti-nuclear and complement C3 levels when compared with PBS. Furthermore, compared with the dsDNA group, the ssDNA group showed significantly lower levels of autoantibody and higher complement C3 (Fig. 6, H to K).

**Fig. 6.**
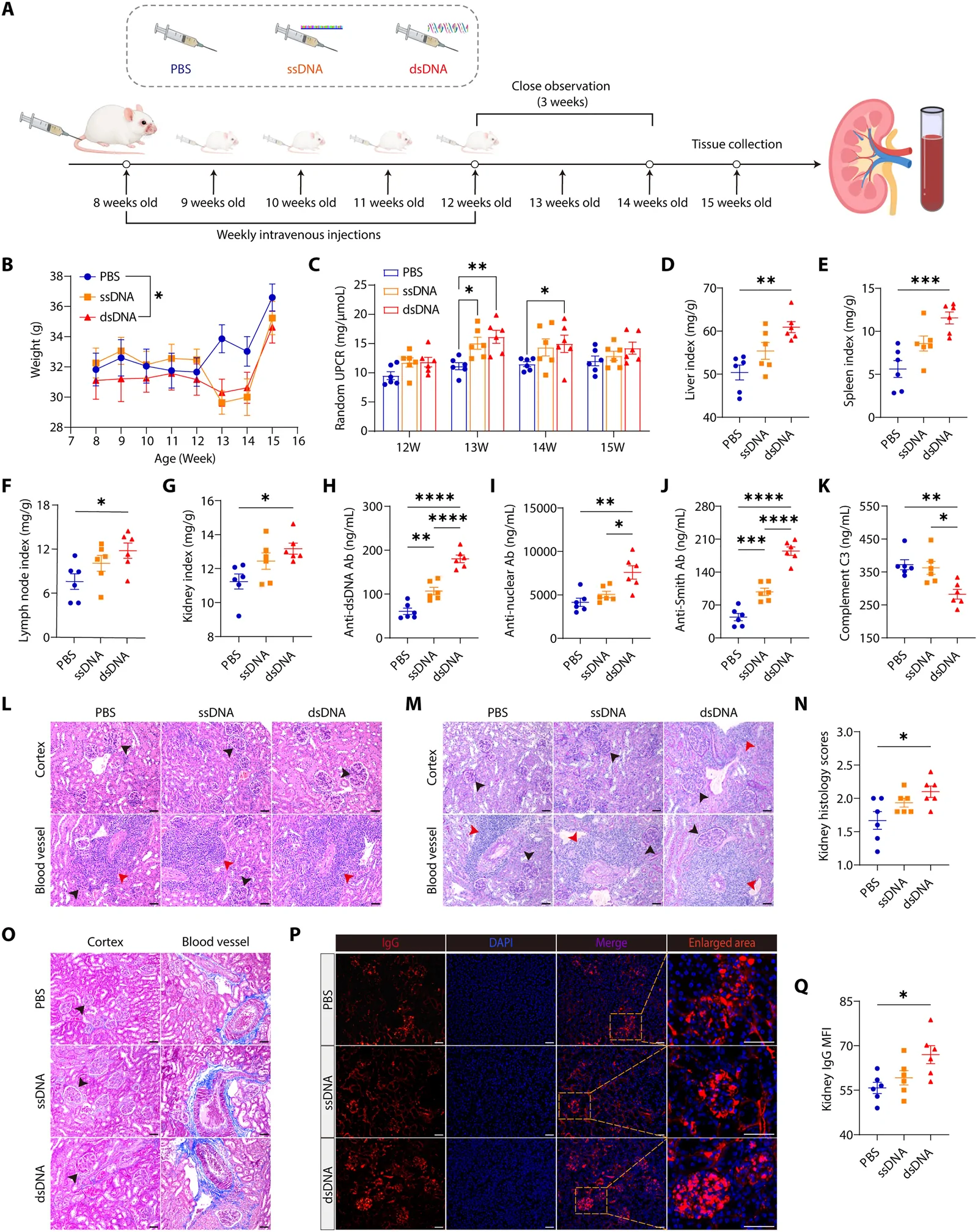
ssDNA probe does not significantly exacerbate lupus-like phenotypes or renal histopathology in MRL/lpr mice. (**A**) Schematic of the exogenous DNA injection intervention in MRL/lpr mice, in which dsDNA was synthesized using the ssDNA sequence from the [^68^Ga]Ga-NOTA-ssDNA probe as a template, followed by weekly injections of PBS, ssDNA, or dsDNA (3 nmol per mouse) starting at 8 weeks for 5 weeks, with 3 weeks of observation, and sacrifice at 15 weeks for organ collection and lupus-like phenotype analysis. (**B**) Body weight change curves of mice in each group (*n* = 6). (**C**) Comparison of random UPCR among groups at 12–15 weeks (*n* = 6). (**D** to **G**) Comparison of liver index (D), spleen index (E), lymph node index (F), and kidney index (G) among groups (*n* = 6). (**H** to **J**) Comparison of plasma anti-dsDNA (H), anti-nuclear (I), and anti-Smith (J) antibody levels among groups (*n* = 6). (**K**) Comparison of plasma complement C3 levels among groups (*n *= 6). (**L** and **M**) Representative H&E-stained (L) and PAS-stained (M) kidney images from PBS, ssDNA, and dsDNA groups, with black arrows indicating glomerular structures and red arrows indicating extensive lymphocyte infiltration; scale bar = 50 μm. (**N**) Comparison of renal histopathological scores among groups (*n* = 6). (**O**) Representative Masson-stained kidney images from each group; scale bar = 50 μm. (**P**) Representative IgG immunofluorescence staining images of kidneys from each group; scale bar = 50 μm. (**Q**) Comparison of mean fluorescence intensity (MFI) of renal IgG deposition among groups (*n *= 6). All data are presented as the mean ± SEM. Statistical analysis for multiple-group comparisons was performed using one-way or two-way ANOVA as appropriate. *P < 0.05, **P < 0.01, ***P < 0.001, ****P < 0.0001.

H&E staining showed glomerular enlargement and perivascular inflammatory cell infiltration in the renal interstitium in all three groups, with more pronounced changes in the dsDNA group compared with PBS and ssDNA groups (Fig. 6L). PAS staining showed a similar trend to H&E staining, with increased mesangial cell proliferation and mesangial matrix expansion in dsDNA group, consistent with pathological features of the acute active phase of LN (Fig. 6M). Integrated histopathological scoring based on the above findings showed that renal pathology scores in the dsDNA group were increased compared with PBS and ssDNA groups, suggesting that exogenous ssDNA administration has limited effect on renal inflammation and structural damage (Fig. 6N). Masson staining showed no significant differences in collagen deposition in glomerular or tubulointerstitial regions across all groups (Fig. 6O). Immunofluorescence staining of IgG deposition in renal tissues showed that IgG-positive fluorescence was observed in the glomeruli of all three groups, with higher intensity and broader distribution in the dsDNA group, consistent with its elevated SLE-specific autoantibody levels and more severe disease phenotype (Fig. 6P). Quantitative analysis of mean fluorescence intensity further showed that IgG fluorescence in renal tissues was significantly increased in the dsDNA group compared with PBS controls. In contrast, IgG fluorescence intensity in the ssDNA group showed no significance when compared with PBS (Fig. 6Q). These findings further indicate that, compared with the strong stimulatory effect of dsDNA on SLE-specific anti-dsDNA antibodies, ssDNA exhibits markedly weaker immunostimulatory activity, suggesting that single or short-term repeated use of the [^68^Ga]Ga-NOTA-ssDNA probe for PET/CT imaging in LN patients is safe and unlikely to exacerbate disease progression.

In summary, compared with dsDNA structures, ssDNA exhibits a significantly safer profile in MRL/lpr mice, offering superior safety advantages in the design of DNA-based radionuclide probes targeting LN.

### Effects of ssDNA and dsDNA on immune cell composition in MRL/lpr mice

To further elucidate the immunological mechanisms underlying the differences in lupus-like phenotypes induced by exogenous ssDNA and dsDNA in MRL/lpr mice, and to verify the safety of the nucleic acid backbone of the [^68^Ga]Ga-NOTA-ssDNA probe, we performed flow cytometry to analyze B-cell and T cell subsets (gating strategy shown in fig. S5).

The proportion of CD19^+^ B cells in the spleen was significantly reduced in both ssDNA and dsDNA groups compared with PBS, with a more pronounced decrease in the dsDNA group (Fig. 7A). In the dsDNA group, activated B cells (CD19^+^CD80^+^PD-L2^+^I-A/I-E^+^IgM^+^IgD^+^), plasmablasts (CD19^+^CD138^+^I-A/I-E^+^IgM^+^), and plasma cells (CD19^−^CD138^+^) were all significantly increased, whereas the ssDNA group showed no significant difference compared with those in PBS group (Fig. 7, B to D). We also examined age-associated B cells (CD19^+^CD11c^+^T-bet^+^) in the spleen, finding that their proportions were significantly increased in both ssDNA and dsDNA groups compared with PBS, with a greater increase in the dsDNA group (Fig. 7E). The proportions of CD3^+^ T cells and double-negative T (DNT), markers of disease progression in MRL/lpr mice (*25*, *26*), increased significantly in the spleen of dsDNA group, but not ssDNA group (Fig. 7, F and G). Regulatory T cell proportions were reduced in both ssDNA and dsDNA groups compared with PBS, with a more marked decrease in the dsDNA group (Fig. 7H). Further analysis of CD4^+^ T cell and CD8^+^ T cell subsets showed that both ssDNA and dsDNA induced differentiation of naive CD4^+^ T cells, characterized by decreased naive and increased non-naive populations (Fig. 7, I and J).

**Fig. 7.**
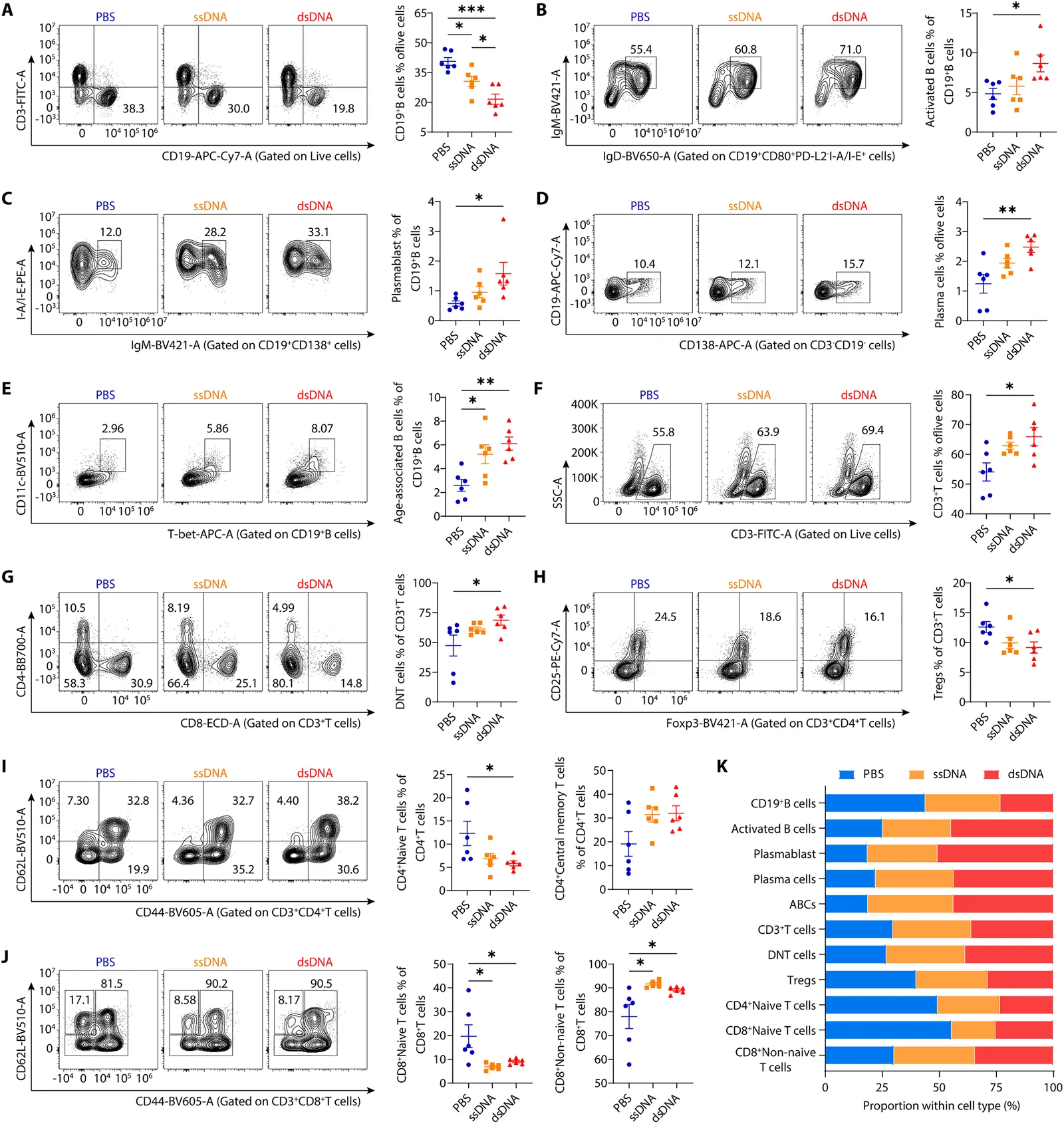
Effects of ssDNA and dsDNA on the composition of immune cell subsets in MRL/lpr mice. (**A**) Representative flow cytometry plots of CD19^+^ B cells in the spleens of mice from each group and quantitative analysis of their frequency among live cells (*n* = 6). (**B** and **C**) Representative flow cytometry plots of activated B cells (B) and plasmablasts (C) in the spleens of mice from each group and quantitative analysis of their frequency among CD19^+^ B cells (*n* = 6). (**D**) Representative flow cytometry plots of splenic plasma cells in mice from each group and quantitative analysis of their frequency among CD3^−^CD19^−^ live cells (*n* = 6). (**E**) Representative flow cytometry plots of age-associated B cells in the spleens of mice from each group and quantitative analysis of their frequency among CD19^+^ B cells (*n* = 6). (**F**) Representative flow cytometry plots of CD3^+^ T cells in the spleens and quantitative analysis of their frequency among live cells (*n* = 6). (**G**) Representative flow cytometry plots of double-negative T (DNT) cells in the spleens and quantitative analysis of their frequency among CD3^+^ T cells (*n* = 6). (**H** and **I**) Representative flow cytometry plots of regulatory T (Treg) cells (H) and CD4^+^ T cell subsets (I) in the spleens and quantitative analysis of their frequency among CD4^+^ T cells (*n* = 6). (**J**) Representative flow cytometry plots of splenic CD8^+^ T cell subsets in mice from each group and quantitative analysis of the frequency of each subset among CD8^+^ T cells (*n *= 6). (**K**) Grouped bar graphs summarizing changes in splenic T- and B-cell subsets across the experimental groups. All data are presented as the mean ± SEM. Statistical analysis for multiple-group comparisons was performed using one-way ANOVA. *P < 0.05, **P < 0.01, ***P < 0.001.

In summary, exogenous DNA stimulation alters the composition of splenic immune cell subsets in MRL/lpr mice, with dsDNA exerting more pronounced effects than ssDNA. dsDNA can exacerbate lupus-like phenotypes by promoting hyperactivation of autoreactive B cells and their terminal differentiation into plasmablasts and plasma cells, inducing expansion of double-negative T cells, and driving the maturation of autoreactive T cells (Fig. 7K). These findings further confirm that ssDNA, as the nucleic acid backbone of the probe, exhibits low immunostimulatory activity and offers superior safety in PET/CT imaging targeting LN.

### dsDNA promotes immune activation via the TLR7/TLR9 signaling pathways in SLE

Previous studies have shown that Toll-like receptor 7 (TLR7) and Toll-like receptor 9 (TLR9) signaling pathways play critical roles in the development of SLE, as they recognize distinct DNA structures and activate downstream IRF5/7 and NF-κB pathways (Fig. 8A), thereby promoting the secretion of inflammatory cytokines such as type I interferons, IL-6, and TNF-α (*27*, *28*). To investigate the molecular mechanisms by which exogenous ssDNA and dsDNA affect the immune microenvironment in SLE, we performed quantitative real-time PCR (RT-PCR) to assess the expression of TLR7/TLR9 pathway-related genes in renal tissues of each group. Compared with the PBS group, mRNA levels of *TLR7*, *TLR9*, *Myd88*, *IRAK4*, *NFκb1*, *IRF7*, *IFNAR1*, and *IFN-*γ were all significantly increased in the dsDNA group, with *TLR9* showing particularly strong upregulation, likely reflecting its preferential recognition of double-stranded DNA. In contrast, ssDNA group only showed increases in *TLR7*, *IRF7*, and *IFNAR1* (Fig. 8, B to I). These findings indicate that dsDNA broadly activates the TLR7/TLR9 signaling pathway and its downstream molecules, whereas ssDNA induces only limited activation. To further validate the functional consequences of these molecular changes, we used cytometric bead array (CBA) and multiplex assays to measure inflammatory cytokines associated with TLR7/TLR9 downstream signaling in plasma. The results showed that, compared with PBS controls, plasma levels of IFN-γ, IL-6, and IL-12p70 were significantly increased in the dsDNA group, while IL-1β, TNF, IL-10, and IL-17A showed upward trends without statistical significance. In contrast, the ssDNA group showed no significant changes in these cytokines (Fig. 8, J to P). These results are consistent with qPCR findings, further confirming that dsDNA strongly activates the TLR7/TLR9 signaling pathway, whereas ssDNA exerts only weak stimulation, insufficient to markedly alter the immune microenvironment in MRL/lpr mice.

**Fig. 8.**
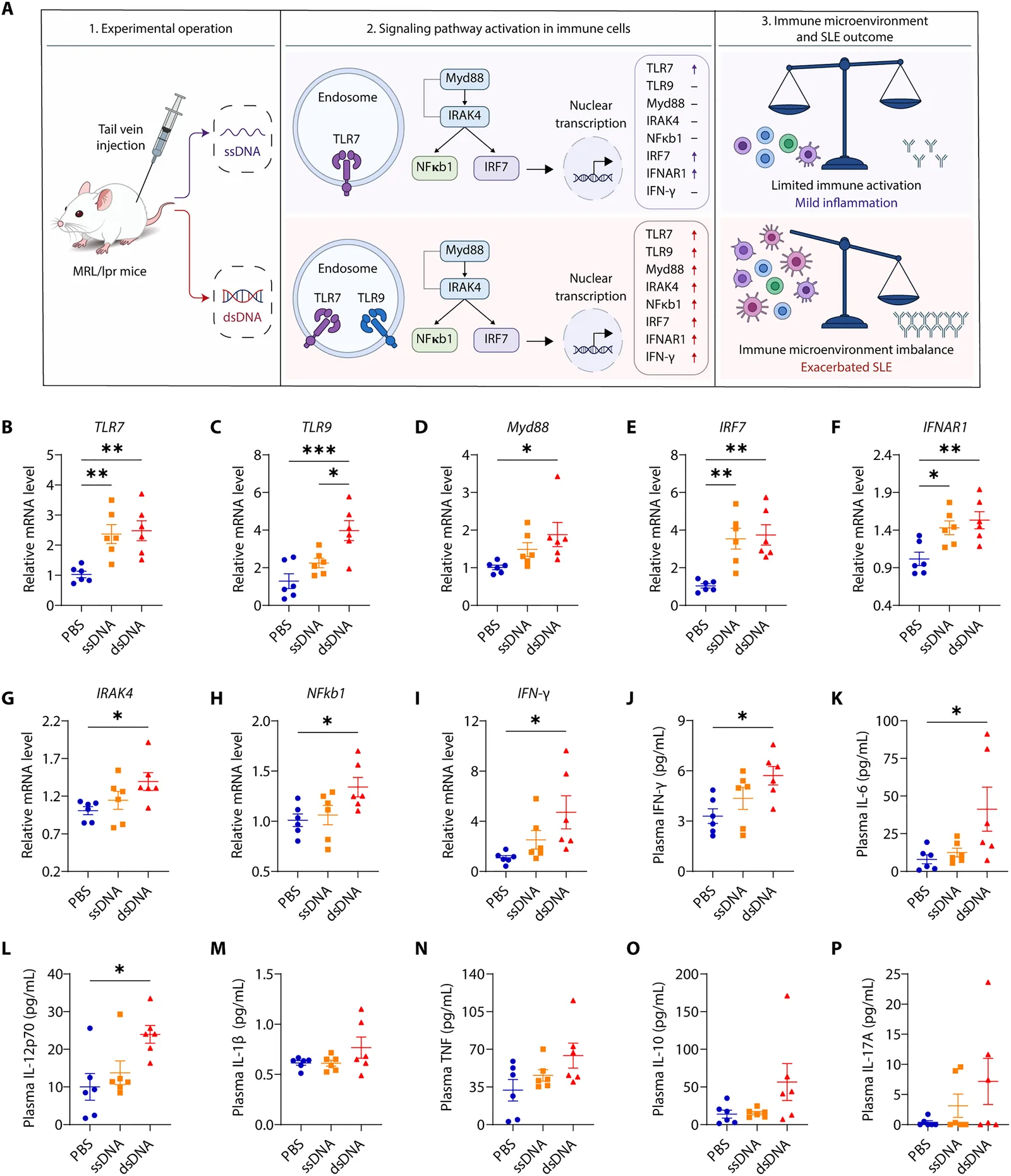
Exogenous DNA regulates immune microenvironment in SLE via the TLR7/TLR9 signaling pathways. (**A**) Schematic overview of exogenous DNA-mediated regulation of the TLR7/TLR9 signaling pathways in the immunopathogenesis of SLE. (**B**) Quantitative real-time PCR analysis of *TLR7* mRNA expression levels in renal tissues across groups (*n* = 6). (**C**) Comparison of *TLR9* mRNA expression levels in renal tissues across groups (*n* = 6). (**D**) Comparison of *Myd88* mRNA expression levels in renal tissues across groups (*n* = 6). (**E**) Comparison of *IRF7* mRNA expression levels in renal tissues across groups (*n* = 6). (**F**) Comparison of *IFNAR1* mRNA expression levels in renal tissues across groups (*n* = 6). (**G**) Comparison of *IRAK4* mRNA expression levels in renal tissues across groups (*n* = 6). (**H**) Comparison of *NFκb1* mRNA expression levels in renal tissues across groups (*n* = 6). (**I**) Comparison of *IFN-*γ mRNA expression levels in renal tissues across groups (*n* = 6). (**J**) Comparison of plasma IFN-γ levels among groups (*n* = 6). (**K**) Comparison of plasma IL-6 levels among groups (*n* = 6). (**L**) Comparison of plasma IL-12p70 levels among groups (*n* = 6). (**M**) Comparison of plasma IL-1β levels among groups (*n* = 6). (**N**) Comparison of plasma TNF levels among groups (*n *= 6). (**O**) Comparison of plasma IL-10 levels among groups (*n* = 6). (**P**) Comparison of plasma IL-17A levels among groups (*n* = 6). All data are presented as the mean ± SEM. Statistical analysis for multiple-group comparisons was performed using one-way ANOVA. *P < 0.05, **P < 0.01, ***P < 0.001.

In summary, exogenous dsDNA exacerbates immune microenvironment imbalance in SLE by activating the TLR7/TLR9 signaling pathways and their downstream inflammatory cytokine networks. In contrast, ssDNA induces only limited activation of this pathway and exhibits low immunostimulatory activity. These findings further validate the safety of selecting ssDNA as the nucleic acid backbone of the [^68^Ga]Ga-NOTA-ssDNA probe in this study.

## DISCUSSION

Early diagnosis of LN is critical to preventing irreversible renal failure in SLE, yet current clinical tools, ranging from invasive biopsies to insensitive serological markers, often fail to detect disease during the nascent stages of immune complex deposition. Although renal biopsy is the gold standard for diagnosis, its invasiveness, associated risks, and inability to be performed repeatedly limit its application in early screening and dynamic monitoring of LN. Commonly used clinical serological markers (such as anti-dsDNA antibodies and complement C3) and urinary biochemical indicators (such as UPCR, serum creatinine, and blood urea nitrogen), although non-invasive, often become abnormal only after significant renal pathological changes have occurred, with insufficient sensitivity and specificity for true early diagnosis (*29*). Therefore, the development of a safe, precise, and repeatable non-invasive diagnostic approach for early LN detection holds significant clinical value.

In this study, we developed a PET/CT radionuclide imaging probe, [^68^Ga]Ga-NOTA-ssDNA, targeting the DNA–anti-dsDNA antibody axis, and systematically evaluated its potential for early non-invasive diagnosis of LN. The results showed that the probe specifically binds anti-dsDNA antibodies in vitro; in the spontaneous LN model (MRL/lpr mice), it accumulates in kidneys with deposited anti-dsDNA antibodies and immune complexes, resulting in prominent and sustained renal radiotracer accumulation on PET/CT images. This enhanced signal was detectable as early as 10 weeks of age, preceding the elevation of conventional renal function markers such as proteinuria, plasma creatinine, and blood urea nitrogen, as well as histopathological evidence of glomerulonephritis, suggesting a meaningful shift in the experimental diagnostic window for LN. Comprehensive safety evaluation further confirmed that the random single-stranded DNA (ssDNA) backbone of the probe does not exacerbate lupus-like phenotypes, disrupt immune cell homeostasis, or activate TLR7/9 signaling pathways in MRL/lpr mice, unlike double-stranded DNA (dsDNA). In summary, the [^68^Ga]Ga-NOTA-ssDNA probe, as a non-invasive, safe, and effective molecular imaging agent, provides a strategy for early LN diagnosis and may address a critical unmet need in SLE clinical management.

The central innovation of our study lies in converting a fundamental immunological marker in LN (anti-dsDNA antibodies) into a clinically quantifiable imaging signal. Anti-dsDNA antibodies are not only LN-specific serological biomarkers but also directly contribute to disease pathogenesis by forming immune complexes with multiple autoantigens that deposit in the glomeruli, triggering complement activation and inflammatory responses. Moreover, these pathogenic autoantibodies can be detected years before the clinical onset of disease (*30*, *31*). Our probe is designed to target these antibodies rather than their antigenic epitopes, thereby effectively leveraging this early pathogenic event. PET/CT imaging with the [^68^Ga]Ga-NOTA-ssDNA probe converts the burden of renal anti-dsDNA antibodies and their immune complex deposition in LN into quantitative imaging parameters, effectively bridging immunopathology and molecular imaging and transforming early diagnosis and disease activity monitoring of LN.

Compared with currently reported PET/CT imaging approaches for LN, such as fluorodeoxyglucose (FDG) PET and fibroblast activation protein inhibitor (FAPI) PET, our [^68^Ga]Ga-NOTA-ssDNA probe operates through a distinct imaging mechanism and offers complementary advantages. FDG PET primarily reflects cellular glucose metabolism and can detect inflammatory cell infiltration in the kidneys, thereby providing the ability to visualize active glomerulonephritis (*32*–*34*). However, the physiological renal excretion of FDG results in high background signals within the renal parenchyma and collecting system, making it difficult to distinguish inflammatory infiltration from tissue repair. In contrast, FAPI PET/CT reflects the expression of fibroblast activation protein and is primarily used to detect tissue fibrosis and remodeling. During the course of LN, FAPI signals may increase before overt histological fibrosis becomes apparent, suggesting its potential for the early detection of renal fibrosis (*35*–*37*). In contrast to these approaches, our probe directly targets pathogenic autoantibodies and immune complexes, enabling the detection of early immunological events before the onset of organ damage or fibrosis while avoiding the nonspecific uptake commonly associated with metabolic tracers. Surface plasmon resonance analysis demonstrated that the binding affinity between ssDNA and anti-dsDNA antibodies was sufficient to support in vivo targeting. This mechanism-specific imaging strategy, which is designed to target pathogenic molecules, is distinct from tracers such as FDG and FAPI that reflect nonspecific inflammatory or fibrotic processes. Importantly, it provides a complementary, mechanism-specific molecular imaging tool by specifically targeting immune complex deposition, the earliest pathogenic event in LN. The combined application of these three tracers may enable a comprehensive assessment of LN pathogenesis from multiple dimensions, including immune complex deposition (upstream), inflammatory activity (intermediate), and fibrosis (downstream), thereby facilitating more accurate disease staging and personalized treatment.

Meanwhile, given that exogenous DNA can act as a danger-associated molecular pattern to exacerbate autoimmunity in SLE (*38*, *39*), we rigorously evaluated the in vivo safety mechanisms of the [^68^Ga]Ga-NOTA-ssDNA probe. We synthesized dsDNA using the ssDNA backbone of the probe as a template and administered repeated injections to 8-week-old MRL/lpr mice for 5 weeks. The results showed that mice in the dsDNA-treated group exhibited significant exacerbation of lupus phenotypes, including hepatosplenomegaly, elevated anti-dsDNA and anti-Smith antibody levels, enhanced B cell activation and plasma cell differentiation, expansion of double-negative T (DNT) cells, activation of the TLR9-MyD88-IRAK4-NFκB signaling pathway, and upregulation of inflammatory cytokines such as IFN-γ and IL-6. In contrast, the ssDNA-treated group showed no significant differences compared with the PBS control group across all parameters, with only slight and transient fluctuations in body weight and proteinuria. These findings confirm that, compared with dsDNA, the ssDNA backbone exhibits weaker immunostimulatory activity. Using ssDNA as the nucleic acid backbone of the probe can largely avoid activation of Toll-like receptor signaling pathways by DNA structures, thereby minimizing the impact of single or repeated imaging on disease progression in LN patients and maximizing imaging safety, laying the foundation for potential clinical translation.

The [^68^Ga]Ga-NOTA-ssDNA PET/CT imaging strategy proposed in this study targets immune complex deposition, a common pathogenic event shared by nearly all active LN subtypes, and therefore could help refine the current paradigm for LN diagnosis and management from multiple perspectives. Currently, renal biopsy remains the gold standard for the diagnosis, classification, and assessment of disease activity in LN. However, its invasiveness, risk of bleeding, and sampling bias limit its utility as a routine monitoring tool. Although PET/CT cannot fully replace renal biopsy for precise histopathological classification, it could serve as a screening tool within a two-step “imaging screening–pathological confirmation” strategy, thereby reducing unnecessary invasive procedures. In patients with newly diagnosed SLE, positive renal immune complex imaging by [^68^Ga]Ga-NOTA-ssDNA PET/CT despite preserved renal function may indicate subclinical LN, thereby helping determine whether immediate renal biopsy is warranted or whether conservative monitoring with serial PET imaging is more appropriate. For patients with established LN, if PET signal intensity correlates well with histological activity indices (such as the activity score in the ISN/RPS classification), noninvasive PET/CT may reduce the need for some repeat renal biopsies for assessing disease remission or guiding the tapering of immunosuppressive therapy. In addition, quantitative changes in PET signal may reflect dynamic changes in immune complex burden before and after treatment. In patients receiving immunosuppressive therapy, a marked reduction in PET signal may indicate therapeutic efficacy even if proteinuria has not yet completely normalized, thereby supporting continuation of the current treatment regimen. Conversely, persistently elevated PET signals, despite stable clinical parameters, may help identify patients at risk of relapse and inform preemptive immunomodulatory interventions, such as increasing glucocorticoid dosage or adding biologic agents, including rituximab. This molecular imaging-based theranostic strategy has the potential to move beyond the traditional symptom-driven treatment paradigm toward precision therapy guided by molecular events. By establishing quantitative models linking PET-derived parameters (such as SUVmax, the kidney-to-liver ratio, or the kidney-to-blood pool ratio) with intrarenal immune complex burden, future studies are required to explore the use of PET signal intensity as a biomarker for stratifying the intensity of immunosuppressive therapy. Patients could be categorized into low-, intermediate-, and high-burden groups corresponding to de-escalation, maintenance, and intensified treatment strategies, respectively, thereby supporting more individualized treatment decisions.

Nevertheless, this study has several limitations, and translating the [^68^Ga]Ga-NOTA-ssDNA probe from the MRL/lpr model to patients with SLE remains challenging. First, the primary data were derived from a single MRL/lpr mouse model. Although this model is widely accepted and recapitulates many features of human SLE, human LN exhibits greater heterogeneity in genetic background, autoantibody profiles, and pathological classifications. Therefore, the general applicability of this probe should be validated in additional lupus models, such as the NZB/W F1 and pristane-induced models, as well as in humanized models. Second, anti-dsDNA antibodies in patients with SLE are highly heterogeneous, with substantial inter-individual variation in epitope recognition, binding affinity, and pathogenicity. Although our probe is based on a DNA scaffold and is therefore theoretically capable of binding multiple anti-DNA antibodies, its applicability across different antibody subclasses (e.g., IgG1, IgG2, and IgG3) and affinity repertoires remains to be established. Third, the safety evaluation of the probe’s nucleic acid backbone was limited to a 5-week repeated administration protocol. Although this protocol preliminarily demonstrated that short-term repeated exposure did not exacerbate the lupus-like phenotype, the potential effects of long-term administration on disease progression require further evaluation over an extended follow-up period. Furthermore, the safety data obtained in MRL/lpr mice cannot be directly extrapolated to humans, and rigorous preclinical toxicology and radiation dosimetry studies will be required before clinical translation. Fourth, the spatial resolution of current PET/CT imaging is insufficient to precisely distinguish immune complex distribution among the glomeruli, renal tubules, and renal interstitium. High-resolution PET/MRI or multimodal imaging technologies may provide more detailed spatial localization in the future, thereby improving the accuracy of renal injury assessment and disease activity monitoring in LN. Finally, the clinical translation of this probe will require overcoming several additional challenges, including standardized large-scale radiotracer production, optimization of imaging dose and acquisition protocols, comprehensive evaluation of human safety and immunogenicity, and validation of its diagnostic performance against renal biopsy as the clinical gold standard.

Based on the above considerations, future studies may proceed along multiple directions. First, iterative optimization of the probe’s oligonucleotide sequence and chelation chemistry could enhance binding affinity and improve its pharmacokinetic properties. For example, aptamer sequences or specific DNA nanostructures with high affinity for anti-dsDNA antibodies or IgG epitopes could be employed to enhance targeting and reduce non-specific interactions (*40*, *41*). Incorporation of linker modifications or PEGylation strategies may help regulate renal clearance and reduce hepatic uptake (*42*, *43*). Second, extending imaging studies to other lupus models and both sexes will help clarify the generalizability of this approach across different lupus phenotypes. Comparative studies with FDG PET and FAPI PET/CT in the same animals could further elucidate the relative sensitivity and specificity of these methods across different LN stages. Additionally, integrating PET/CT imaging data with serological tests and emerging biomarkers could enable the development of multiparametric diagnostic models for precise stratification and personalized treatment of LN. Third, the ssDNA sequence of the [^68^Ga]Ga-NOTA-ssDNA probe could be adapted as a backbone for theranostic platforms by replacing ^68^Ga with therapeutic radionuclides (e.g., ^177^Lu) to develop radioligand therapy (*44*). This strategy could enable both early diagnostic imaging and targeted radiation delivery to pathogenic immune complexes, offering therapeutic options for refractory LN. Fourth, after sufficient preclinical data have been accumulated, clinical translation of this probe in LN should be explored. Initial clinical translation would begin with phase 1 trials in healthy volunteers to assess safety, biodistribution, and dosimetry, followed by phase 2 studies in SLE patients with or without renal involvement. If proven safe and effective, the probe could be integrated into LN diagnostic and monitoring workflows, correlating renal PET signals with histological activity indices from biopsy to reduce the need for repeated biopsies and guide personalized treatment. Fifth, given that autoantibodies such as anti-dsDNA also play key roles in other immune complex–mediated diseases (e.g., ANCA-associated vasculitis and membranous nephropathy) (*45*, *46*), exploring similar imaging strategies may have important translational value for diagnosis and monitoring of these conditions.

In summary, this study demonstrates that molecular imaging targeting the DNA–anti-dsDNA antibody axis represents a highly promising strategy for early, non-invasive diagnosis of LN. While current practice relies on indirect, systemic biomarkers (e.g., serum anti-dsDNA) or renal biopsy, which is invasive and usually performed at a stage with significant renal damage, the [^68^Ga]Ga-NOTA-ssDNA probe we developed images the site-specific pathological event itself directly: renal deposition of pathogenic immune complexes at a preclinical stage before organ damage occurs. It offers advantages for LN diagnosis, including early detection, non-invasiveness, safety, and suitability for non-invasive monitoring of disease activity/therapeutic effects. Beyond diagnosis, this probe has the potential to support the entire continuum of LN management—from early screening, precise disease classification, relapse prediction, and treatment response assessment to patient selection for targeted therapies and even radioligand therapy—thereby shifting LN management from a traditional symptom- and laboratory marker-driven approach toward a precision medicine paradigm guided by molecular events, ultimately providing a framework for the clinical management of SLE.

## MATERIALS AND METHODS

### Animals

Female MRL/lpr mice used in this study were purchased from Cyagen Biosciences (Suzhou, China), and age-matched female control mice were obtained from Hubei Biente Biotechnology Co., Ltd. All mice were housed in a specific pathogen-free (SPF) facility at the Animal Centers at Fujian Medical University Union Hospital and Tongji Hospital of Tongji Medical College of Huazhong University of Science and Technology, under a 12 h light/dark cycle, with humidity maintained at 40–70% and temperature at 20–26°C. No more than five mice were housed per cage, with ad libitum access to food and water. All animal procedures were approved by the Animal Ethics Committees at Fujian Medical University Union Hospital (Approval No. IACUC FJMU 2025–0377 and 2025–0170) and Tongji Hospital of Tongji Medical College of Huazhong University of Science and Technology (Approval No. TJH-202411001) and complied with the guidelines for the care and use of laboratory animals of Huazhong University of Science and Technology.

Throughout the study, mouse body weight was monitored weekly, and urine samples were collected. At designated time points (6, 10, 12, and 16 weeks of age), mice were euthanized by CO2 inhalation, and plasma as well as tissues including liver, spleen, kidney, heart, and lung were collected for subsequent analyses. The liver, spleen, lymph node, and kidney indices were calculated as the ratio of the respective organ weight (mg) to body weight (g).

### Assessment of dermatitis

As MRL/lpr mice age, spontaneous inflammatory skin lesions develop on the forehead, ears, and dorsocervical region. A semi-quantitative dermatitis score (0–3) was assigned based on the extent of skin involvement in these areas: 0: no obvious skin changes; 1: mild alopecia with erythema and a few scattered lesions; 2: erythema, crusting, and alopecia with localized involvement; 3: ulceration with extensive involvement. Detailed scoring criteria are described in previous studies (*47*).

### Measurement of autoantibody and complement C3 levels

Enzyme-linked immunosorbent assay (ELISA) was used to measure the levels of anti-double-stranded DNA antibodies, antinuclear antibodies, anti-Smith antibodies, and complement C3 in mouse plasma. Commercial ELISA kits were used according to the manufacturer’s instructions: mouse anti-dsDNA antibody (IgG) ELISA kit (Cat#CSB-E11194m, CUSABIO, https://www.cusabio.com/), mouse antinuclear antibody (IgG) ELISA kit (Cat#CSB-E12912m, CUSABIO), mouse anti-Smith antibody ELISA kit (Cat#CSB-E15976m, CUSABIO) and mouse complement C3 ELISA kit (Cat#E-EL-M0330, Elabscience). All samples were measured in duplicate, and antibody concentrations were calculated from standard curves using Curve Expert software.

### Evaluation of renal function damage

Creatinine levels in mouse urine and plasma were measured using a creatinine assay kit (sarcosine oxidase method, Nanjing Jiancheng Bioengineering Institute, Cat#C011–2-1). Blood urea nitrogen (BUN) levels in mice were measured using a commercial assay kit (Nanjing Jiancheng Bioengineering Institute, Cat#C013–2-1). Total protein concentration in mouse urine was quantified using a BCA protein assay kit (BOSTER, Cat#AR0197). UPCR was calculated as urinary protein concentration divided by urinary creatinine concentration to assess early renal function impairment.

### Pathological staining and histological scoring of the kidney

The right kidney of each mouse was fixed in 4% paraformaldehyde, embedded in paraffin, and sectioned into 4 μm slices. Sections were subjected to hematoxylin and eosin (H&E), periodic acid-Schiff (PAS), and Masson staining to evaluate glomerular structural damage, mesangial alterations, and renal fibrosis. Renal histopathological changes were evaluated using a semi-quantitative scoring system. Fifteen glomeruli per mouse were randomly selected and averaged, with scores ranging from 0 to 3: 0: no change; 1: mild cellular proliferation or infiltration; 2: mesangial proliferation, hyaline deposition, or lobular changes; 3: severe cellular proliferation/infiltration, diffuse hyalinosis, or crescent formation (*48*). All scoring was performed in a blinded manner.

### Immunofluorescence of renal immune complex deposition

Deparaffinized kidney sections were subjected to direct immunofluorescence staining using DAPI and YSFluor 594 goat anti-mouse IgG (H + L) secondary antibody (Yeasen, Cat#33212ES60, 1:300) to detect IgG immune complex deposition in glomeruli. Images were acquired at 20× magnification using an Olympus BX53 upright fluorescence microscope. Immunofluorescence intensity was quantitatively analyzed using ImageJ software (V1.8) under blinded conditions. At least 10 glomeruli per mouse were analyzed to calculate the mean fluorescence intensity (MFI) of renal tissue for each mouse.

### Flow cytometry analysis of the spleen

The harvested mouse spleens were mechanically dissociated, filtered, and treated with red blood cell lysis buffer (Solarbio, Cat#R1011) to obtain single-cell suspensions, and viable cells were counted using trypan blue staining. A total of 1 × 10^6^ cells were used for flow cytometry staining. All staining procedures were performed at 4°C. Cells were first incubated with Fc blocking reagent TruStain FcX PLUS (anti-mouse CD16/32 antibody; BioLegend, Cat#156603, 1:100) in FACS buffer (PBS containing 0.5% BSA, 2 mM EDTA, and 0.1% sodium azide) to block Fc receptors. After blocking, cells were stained with Fixable Viability Stain 700 (BD, Cat#564997, 1:1000) to distinguish live and dead cells for gating. After staining, cells were washed once with PBS and then subjected to the following surface and intracellular staining procedures for flow cytometry (*49*).

Cells were incubated with the following antibodies according to the pre-designed flow cytometry surface staining panel at 4°C for 30 min: FITC anti-mouse CD3 (BioLegend, Cat#100204, 1:100), APC/Cy7 anti-mouse CD19 (BioLegend, Cat#115530, 1:100), APC anti-mouse CD138 (Syndecan-1) (BioLegend, Cat#142506, 1:100), Brilliant Violet 421 anti-mouse IgM (BioLegend, Cat#406532, 1:100), Brilliant Violet 650 anti-mouse IgD (BioLegend, Cat#405721, 1:100), PE anti-mouse I-A/I-E (BioLegend, Cat#107608, 1:100), PE/Cy7 anti-mouse CD273 (B7-DC, PD-L2) (BioLegend, Cat#107214, 1:100), PerCP/Cy5.5 anti-mouse CD80 (BioLegend, Cat#104722, 1:100), BD Horizon BB700 Rat Anti-Mouse CD4 (BD, Cat#566407, 1:100), BD Horizon PE-CF594 Rat Anti-Mouse CD8a (BD, Cat#562315, 1:100), Brilliant Violet 605 anti-mouse/human CD44 (BioLegend, Cat#103047, 1:100), and Brilliant Violet 510 anti-mouse CD62L (BioLegend, Cat#104441, 1:100). After staining, cells were washed twice with PBS, resuspended in FACS buffer, and analyzed using a 13-color Beckman CytoFLEX flow cytometer. Data were analyzed using FlowJo software (V10.8.1).

Intracellular transcription factor staining protocol: After viability staining, surface staining was first performed using the following antibodies: FITC anti-mouse CD3 (BioLegend, Cat#100204, 1:100), APC/Cy7 anti-mouse CD19 (BioLegend, Cat#115530, 1:100), BD Horizon BB700 Rat Anti-Mouse CD4 (BD, Cat#566407, 1:100), BD Horizon PE-CF594 Rat Anti-Mouse CD8a (BD, Cat#562315, 1:100), and PE/Cy7 anti-mouse CD25 (BioLegend, Cat#102016, 1:100). After incubation at 4°C for 30 min, cells were washed twice with PBS. Then, 1 mL of fixation/permeabilization working solution (Invitrogen eBioscience Foxp3/Transcription Factor Staining Buffer Set, Cat#00–5523-00) was added, vortexed, and incubated at 4°C in the dark for 45 min, followed by centrifugation and removal of the supernatant. Cells were then stained overnight at 4°C in the dark with intracellular transcription factor antibodies: Brilliant Violet 421 anti-mouse FOXP3 (BioLegend, Cat#126419, 1:100) and APC anti-T-bet (BioLegend, Cat#644814, 1:100). After staining, cells were washed twice with 1× permeabilization buffer, resuspended in FACS buffer, and analyzed using a 13-color Beckman CytoFLEX flow cytometer. Data were analyzed using FlowJo software (V10.8.1).

### Plasma cytokine and chemokine detection

Mouse plasma was obtained via blood collection from the retro-orbital venous plexus. Collected blood was placed into EDTA-coated anticoagulant tubes and centrifuged at 1000 g for 20 min at 4°C to separate plasma. Plasma cytokine levels were measured using the BD Cytometric Bead Array (CBA) Mouse Th1/Th2/Th17 kit (BD, Cat#560485) and the ABplex Mouse 15-Plex Panel ELISA kit (Abclonal, Cat#RK05203). All procedures were performed strictly according to the manufacturers’ instructions. The cytokines analyzed included IFN-γ, IL-2, IL-4, IL-5, IL-6, IL-10, IL-12p70, IL-17A, and TNF.

### RNA extraction and quantitative real-time PCR (qRT-PCR)

Total RNA was extracted from mouse kidney tissues using TRIzol reagent (Invitrogen, Cat#15596018), and cDNA was synthesized by reverse transcription according to the instructions of the Hifair III 1st Strand cDNA Synthesis Kit (Yeasen, Cat#11139ES60). Quantitative real-time PCR was performed using Hieff qPCR SYBR Green Master Mix (Yeasen, Cat#11201ES60) on a Quantagene q225 real-time PCR system (Kubo Technology, Beijing, China) according to the manufacturer’s instructions, and melting curves and cycle threshold (Ct) values were obtained. Glyceraldehyde-3-phosphate dehydrogenase (GAPDH) was used as an internal reference gene to normalize target gene expression and calculate ΔCt values, and relative mRNA expression levels were determined using the 2^–ΔΔCt^ method. The primer sequences used in this study are listed in table S2.

### Surface plasmon resonance (SPR) analysis

The binding affinity between the random single-stranded DNA sequence in the [^68^Ga]Ga-NOTA-ssDNA probe and anti-dsDNA IgG antibody (Novus Biologicals, Cat#NBP3–07302) was measured using surface plasmon resonance (SPR) on a Biacore T200 biomolecular interaction analysis system (GE Healthcare, USA). Anti-dsDNA IgG antibody (100 nM) was dissolved in acetate buffer (pH 5.0) and immobilized on a CM5 sensor chip (GE Healthcare, USA) via amine coupling. Subsequently, single-stranded DNA solutions at different concentrations (0–5 μM, in PBS) were injected over the chip surface at a flow rate of 30 μL/min to monitor association and dissociation in real time. The resulting sensorgrams were fitted using Biacore T200 evaluation software based on a 1:1 Langmuir binding model. The equilibrium dissociation constant (KD) and other kinetic parameters were calculated using Biacore Insight Evaluation software. All experiments were conducted at 25°C, with reference channel signals and buffer blanks subtracted to correct for non-specific binding.

### Construction of [^68^Ga]Ga-NOTA-ssDNA probe and radiotracer formulation

NOTA-A20 and T20-ssDNA oligonucleotides were purchased from Sangon Biotech (Shanghai, China). The nucleotide sequence of T20-ssDNA was 5′-TTTTTTTTTTTTTTTTTTTTTTAGCGACGTACGATCGTACGACT-3′. The lyophilized T20-ssDNA oligonucleotide was dissolved in nuclease-free water and vortexed and centrifuged to prepare a 100 μM stock solution.

The preparation of the [^68^Ga]Ga-NOTA-ssDNA probe was as follows: 1 mL of [^68^Ga]GaCl_3_ solution was eluted from a ^68^Ge/^68^Ga generator (ITM Medical Isotopes GmbH, Germany) using 0.05 M HCl. A total of 74 MBq of [^68^Ga]GaCl3 was taken, and the pH was adjusted to 4.0–4.5 using 0.25 M sodium acetate. Then, 30 μL of NOTA-A20 (100 μM, 3 nmol) was added, and the mixture was incubated at 37°C with shaking at 400 rpm for 30 min. After the reaction, [^68^Ga]Ga-NOTA-A20 was purified using a PD-10 desalting column with phosphate-buffered saline as the mobile phase, followed by sterile filtration through a 0.22 μm filter (Millipore, USA). The purified [^68^Ga]Ga-NOTA-A20 was mixed with T20-ssDNA at a 1:1 molar ratio in phosphate buffer and incubated at 37°C with shaking for 15 min to allow hybridization, yielding the [^68^Ga]Ga-NOTA-ssDNA probe. The final product was filtered through a 0.22 μm membrane into sterile vials for storage. Size exclusion chromatography (SEC) was used to assess radiochemical yield (RCY) and radiochemical purity (RCP), using a BioCore SEC-150 column (5 μm, 7.8 × 300 mm, NanoChrom).

### Dynamic micro PET/CT imaging studies

Dynamic PET/CT imaging was performed on control and MRL/lpr mice at 6, 10, 12, and 16 weeks of age using an Inveon MM micro-PET/CT scanner (Siemens Ltd., Munich, Germany). Prior to imaging, mice were anesthetized with 2% isoflurane and injected via the tail vein with [^68^Ga]Ga-NOTA-ssDNA (approximately 100–200 μL per mouse, containing 3.7 MBq radioactivity and 0.5 nmol probe), followed by continuous 60-minute dynamic scanning in the prone position. Data were acquired in list mode and reconstructed into 20 frames (6 × 10 s, 9 × 60 s, and 5 × 600 s). PET images were reconstructed using ordered subset expectation maximization 3D or maximum a posteriori algorithms (OSEM3D/MAP) with attenuation correction. Regions of interest (ROIs) were drawn on attenuation-corrected whole-body coronal images using Inveon Research Workplace software (Siemens, Germany) for major organs (heart, liver, kidney, etc.), and both mean and maximum %ID/g values were calculated.

To generate time-activity curves for ROIs, radiotracer uptake at each time point was expressed as %ID/g. ROI data were used to analyze the distribution and clearance characteristics of the probe in the kidneys and other major organs, and curve fitting was performed using Prism 9.0 software (GraphPad, USA) to generate corresponding kinetic equations.

### Probe target specificity test and competitive blockade experiment

To evaluate the target specificity of the probe and its colocalization with immune complexes in renal tissue, 16-week-old mice were assigned to three groups: Control, MRL/lpr, and MRL/lpr mice pre-injected with an excess of unlabeled ssDNA (blocking group). Unlabeled ssDNA was administered via the tail vein at 50 nmol/g 10 minutes prior to probe injection to saturate available binding sites. Cy5.5-labeled ssDNA (Cy5.5-ssDNA) was then injected intravenously via the tail vein at a dose of 0.5 nmol per gram body weight. At 60 min postinjection, mice were euthanized, and the kidneys were harvested, embedded in OCT compound, snap-frozen, and cryosectioned at 4 μm. Frozen sections were fixed in 4% PFA, blocked with 0.5% BSA, and incubated overnight at 4°C in the dark with YSFluor 594-conjugated goat anti-mouse IgG (H + L) secondary antibody (Yeasen, Cat# 33212ES60; 1:300) to detect IgG deposited in the renal tissue. Nuclei were counterstained with DAPI. Fluorescence images were acquired under identical acquisition settings across all groups. The spatial correspondence between the Cy5.5-ssDNA signal and the IgG signal was analyzed in ImageJ (v1.8) using the 3D Surface Plot function to assess the specific accumulation of the probe at sites of glomerular immune-complex deposition.

### In vivo and ex vivo fluorescence imaging of Cy5.5-ssDNA in control and MRL/lpr mice at different ages

A Cy5.5-labeled ssDNA probe (Cy5.5-ssDNA) was administered to Control and MRL/lpr mice of different ages via tail vein injection at a dose of 0.1 nmol/g. In vivo fluorescence imaging was performed at 30, 45, and 60 min after injection (excitation/emission wavelengths, 754/778 nm). After the final imaging session, mice were euthanized, and the heart, liver, spleen, kidneys, and other major organs were harvested for ex vivo fluorescence imaging.

### Biodistribution analysis

After dynamic PET/CT imaging, the imaged mice (*n* = 4) were euthanized by CO_2_ inhalation, and blood samples were immediately collected from the retro-orbital venous plexus using capillary tubes. The mice were then dissected, and major organs and tissues (including brain, heart, lungs, liver, spleen, kidneys, stomach, small intestine, large intestine, muscle, bone, and tail) were collected, weighed, and their radioactivity was measured using an automated gamma counter (radioimmunoassay analyzer). Radioactive uptake was expressed as a percentage of injected dose per gram of tissue (%ID/g), calculated as tissue radioactivity (cpm/g) divided by total injected radioactivity (cpm) multiplied by 100%.

### Fitting of the renal dynamic uptake–clearance–retention model and extraction of pharmacokinetic parameters

Dynamic renal PET signals were expressed as radiotracer uptake (%ID/g) within the region of interest (ROI). Time-activity curves (TACs) from control and MRL/lpr mice at 6, 10, 12, and 16 weeks of age were analyzed separately, and data at each time point are presented as the group mean ± standard error of the mean (SEM).

To characterize the uptake, subsequent clearance, and persistent signal observed in renal time–activity curves (TACs), empirical uptake–clearance (UC) and uptake–clearance–retention (UCR) models were evaluated. The UCR model extends the UC model by incorporating an additional component to describe sustained renal tracer signal.

The UC model was defined as

C(t)=A(e−kcleart−e−kuptaket) (1)

where *A* represents the overall amplitude of the uptake–clearance process, *k_uptake_* is the apparent uptake rate constant, and *k_clear_* is the apparent clearance rate constant. The model satisfies the boundary condition C(0) = 0 and describes the uptake and subsequent decline of the renal tracer signal.

The UCR model incorporates an additional retention term into the UC model

$$C\left(t\right)=A\left(e^{-k_{\text{clear}}t}-e^{-k_{\text{uptake}}t}\right)+R\left(1-e^{-k_{\text{retention}}t}\right)$$ (2)

where *R* represents the apparent capacity of the disease-associated retention component, and *k_retention_* is the apparent formation rate constant of the retained component. This model was used to describe the persistent or plateau-like renal PET signal observed in late-stage MRL/lpr mice.

Model fitting was performed by bounded nonlinear least-squares optimization using the trust-region reflective (TRF) algorithm implemented in SciPy. To avoid overfitting, model selection was performed using the small-sample corrected Akaike information criterion (AICc). The UC and UCR models contained three and five nominal parameters, respectively. If the UC model yielded a lower AICc, R was set to zero, indicating that the retention term was unnecessary. Goodness-of-fit was evaluated using the coefficient of determination (R^2^), AICc, and the root mean square error (RMSE).

After successful model fitting, core pharmacokinetic parameters were calculated from the renal TACs of each group using non-compartmental analysis, including peak uptake (C_max_), time to peak (T_max_), the area under the time-activity curve from 0–60 min (AUC_0–60 min_), the ratio of the 60-min signal to the peak signal (C60/C_max_), and the apparent tissue clearance half-life.

AUC_0–60 min_ was calculated using the trapezoidal rule. C60/C_max_ was used to quantify the fraction of peak signal retained at 60 min. The apparent tissue clearance half-life was estimated from the log-linear portion of the TAC between 15 and 60 min

$$k_{\mathrm{app}}=-\text{slope}\left[\text{log}C\left(t\right)\right]$$ (3)

$$t_{1/2,\mathrm{app}}=\frac{\text{ln}2}{k_{\mathrm{app}}}$$ (4)

When the terminal portion of the TAC did not reach a stable log-linear clearance phase (terminal log-linear R^2^ < 0.9), or when the estimated half-life exceeded the 60-min observation window, the value was reported as “>60 min (unreliable estimate)” and was not interpreted as the true terminal elimination half-life.

### Statistical analysis

All data in this study were analyzed using GraphPad Prism 9 software, except for renal TAC modeling and related computational analyses, which were performed in Python 3.10.18 using NumPy 2.2.6, pandas 2.3.1, SciPy 1.15.3, Matplotlib 3.10.3, and openpyxl 3.1.5. Quantitative data are presented as mean ± standard error of the mean (Mean ± SEM). All statistical tests were two-tailed. For comparisons between two groups, if the data were normally distributed with equal variances, an unpaired two-tailed Student’s *t* test was used. If variances were unequal, Welch’s corrected *t* test was applied. For comparisons among multiple groups, one-way ANOVA or two-way ANOVA followed by post-hoc tests was used when data met assumptions of normality and equal variance. If the data were not normally distributed, the Mann-Whitney U test was used for comparisons between two groups. Correlations between two variables were analyzed using simple linear regression. Pearson’s correlation coefficient (r) and the corresponding 95% confidence interval (CI) were calculated and reported. *P* < 0.05 was considered statistically significant (*P < 0.05; **P < 0.01; ***P < 0.001; ****P < 0.0001).
