# Supplementary material for: Pathology-targeted PET/CT imaging of early lupus nephritis via a 68Ga-labeled single-stranded DNA probe
Source: Sci Adv. 2026 Nov 13;12(46):eaei3589. doi: 10.1126/sciadv.aei3589 (PMC13763964; doi:10.1126/sciadv.aei3589)
Supplement: Supplementary file 1 [file sciadv.aei3589_sm.pdf]

Science Advances
AAAS

Supplementary Materials for

Pathology-targeted PET/CT imaging of early lupus nephritis via a ^{68}Ga-labeled single-stranded DNA probe

Zhiwen Huang *et al.*

Corresponding author: Xiaoquan Rao, jannet18@163.com; Dawei Jiang, daweijiang@hust.edu.cn;
Jixin Zhong, zhongjixin620@163.com

Sci. Adv. **12**, eaei3589 (2026)
DOI: 10.1126/sciadv.aei3589

This PDF file includes:

Figs. S1 to S5
Tables S1 and S2

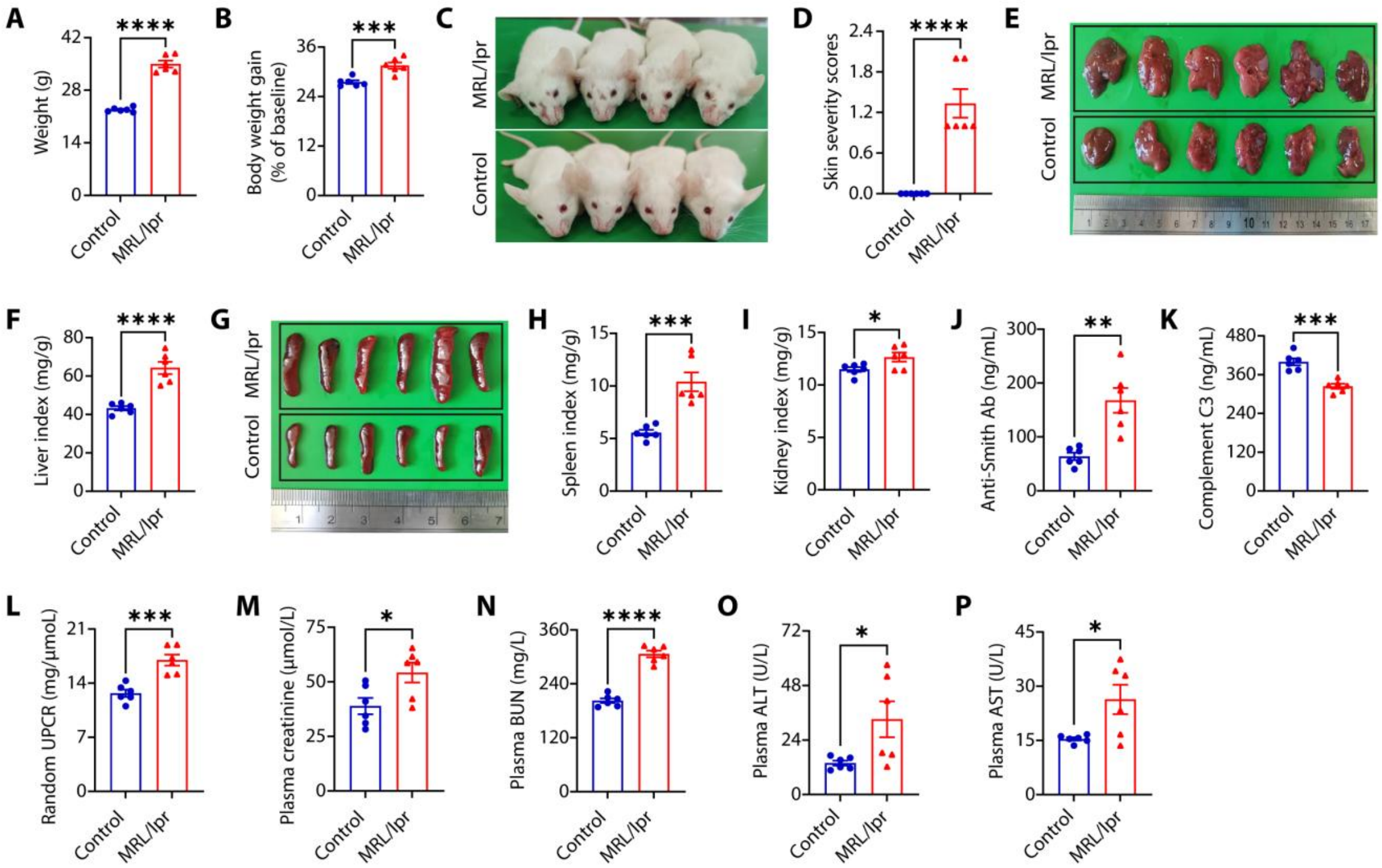

Fig. S1. MRL/lpr mice spontaneously develop pronounced lupus-like alterations at the age of 16 weeks. (A) Comparison of body weight between 16-week-old Control and MRL/lpr groups (n = 6). (B) Comparison of body weight gain relative to the 6-week baseline between 16-week-old Control and MRL/lpr groups (n = 6). (C) Representative gross images of facial and dorsocervical skin in 16-week-old Control and MRL/lpr mice. (D) Comparison of lupus-like skin lesion scores between 16-week-old Control and MRL/lpr groups (n = 6). (E) Representative gross images of the liver in 16-week-old Control and MRL/lpr groups. (F) Comparison of liver indices between 16-week-old Control and MRL/lpr groups (n = 6). (G) Representative gross images of the spleen in 16-week-old Control and MRL/lpr groups. (H) Comparison of spleen indices between 16-week-old Control and MRL/lpr groups (n = 6). (I) Comparison of renal indices between 16-week-old Control and MRL/lpr groups (n = 6). (J) Comparison of plasma anti-Smith antibody levels between 16-week-old Control and MRL/lpr groups (n = 6). (K) Comparison of plasma complement C3 levels between 16-week-old Control and MRL/lpr groups (n = 6). (L) Comparison of random UPCR between 16-week-old Control and MRL/lpr groups (n = 6). (M) Comparison of plasma creatinine levels between 16-week-old Control and MRL/lpr groups (n = 6). (N) Comparison of plasma blood urea nitrogen levels between 16-week-old Control and MRL/lpr groups (n = 6). (O) Comparison of plasma alanine aminotransferase (ALT) levels between 16-week-old Control and MRL/lpr groups (n = 6). (P) Comparison of plasma aspartate aminotransferase (AST) levels between 16-week-old Control and MRL/lpr groups (n = 6). All data are presented as the mean ± SEM. Statistical

analysis between two groups was performed using a two-tailed unpaired Student's t-test. *$P < 0.05$, **$P < 0.01$, ***$P < 0.001$, ****$P < 0.0001$.

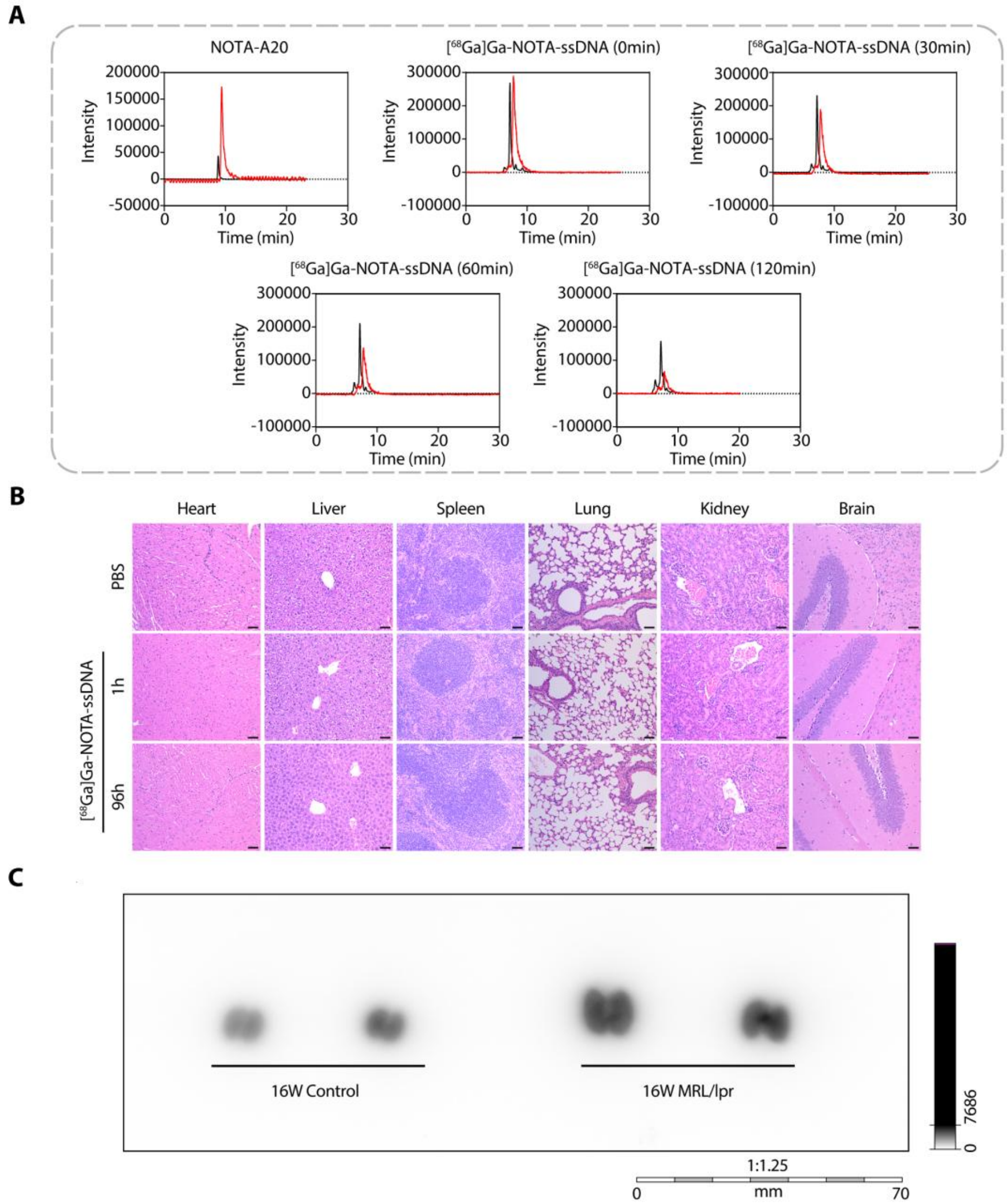

Fig. S2. Characterization of the [^{68}Ga]Ga-NOTA-ssDNA probe and ex vivo autoradiography in 16-week-old MRL/lpr mice with lupus nephritis (LN). (A) Structural stability of NOTA-A20 and the [^{68}Ga]Ga-NOTA-ssDNA probe at 0, 30, 60, and 120 minutes after synthesis assessed by high-performance liquid chromatography (HPLC). (B) Representative H&E-stained images of major organs at 1 and 96 hours after injection of the [^{68}Ga]Ga-NOTA-ssDNA probe into BALB/c mice; scale bar = 50 µm. (C) Representative ex vivo autoradiography

images of kidneys from 16-week-old control and MRL/lpr mice after administration of [^{68}Ga]Ga-NOTA-ssDNA.

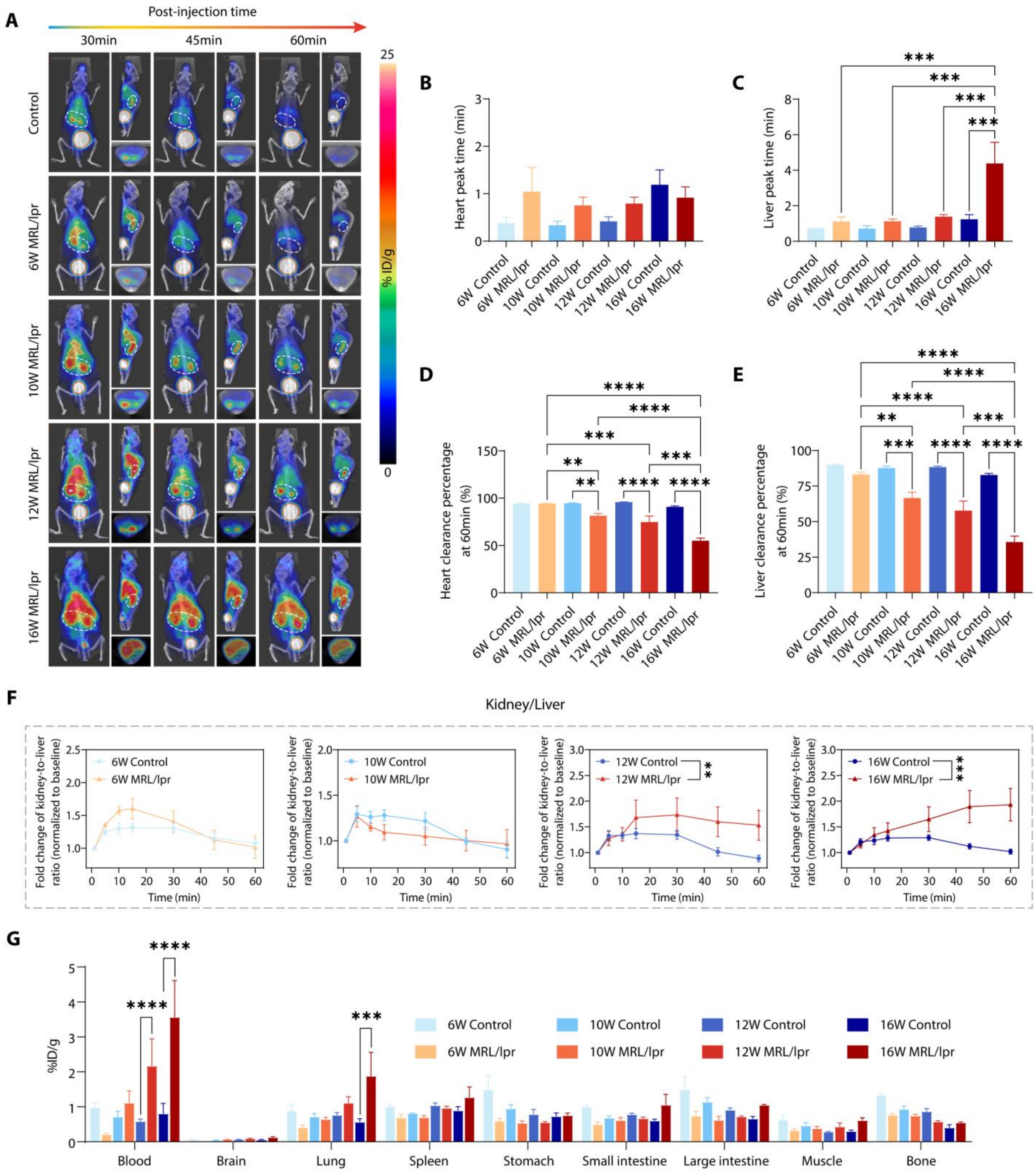

Fig. S3. PET/CT imaging characteristics and ex vivo biodistribution of the [^{68}Ga]Ga-NOTA-ssDNA probe in LN across different ages. (A) Longitudinal monitoring of maximum intensity projection (MIP) images from dynamic PET/CT scans acquired with [^{68}Ga]Ga-NOTA-ssDNA in MRL/lpr mice at different ages (kidney regions indicated by white dashed circles). (B) Comparison of time to peak cardiac uptake of the [^{68}Ga]Ga-NOTA-ssDNA probe in Control and MRL/lpr mice at 6, 10, 12, and 16 weeks of age (n = 4). (C) Comparison of time to peak hepatic uptake of the [^{68}Ga]Ga-NOTA-ssDNA probe in Control and MRL/lpr mice at 6, 10, 12, and 16 weeks of age (n = 4). (D) Comparison of cardiac clearance rates at

60 minutes post-injection of the [^{68}Ga]Ga-NOTA-ssDNA probe in Control and MRL/lpr mice at 6, 10, 12, and 16 weeks of age. (E) Comparison of hepatic clearance rates at 60 minutes post-injection of the [^{68}Ga]Ga-NOTA-ssDNA probe in Control and MRL/lpr mice at 6, 10, 12, and 16 weeks of age (n = 4). (F) Time-dependent fold-change curves of kidney-to-liver uptake ratios within 60 minutes after injection of the [^{68}Ga]Ga-NOTA-ssDNA probe in Control and MRL/lpr mice at different ages (n = 4). (G) Ex vivo biodistribution analysis of the [^{68}Ga]Ga-NOTA-ssDNA probe in selected organs at 60 minutes post-injection in Control and MRL/lpr mice of different ages. All data are presented as the mean ± SEM. Statistical analysis for multiple-group comparisons was performed using one-way or two-way ANOVA as appropriate. ***P* < 0.01, ****P* < 0.001, *****P* < 0.0001.

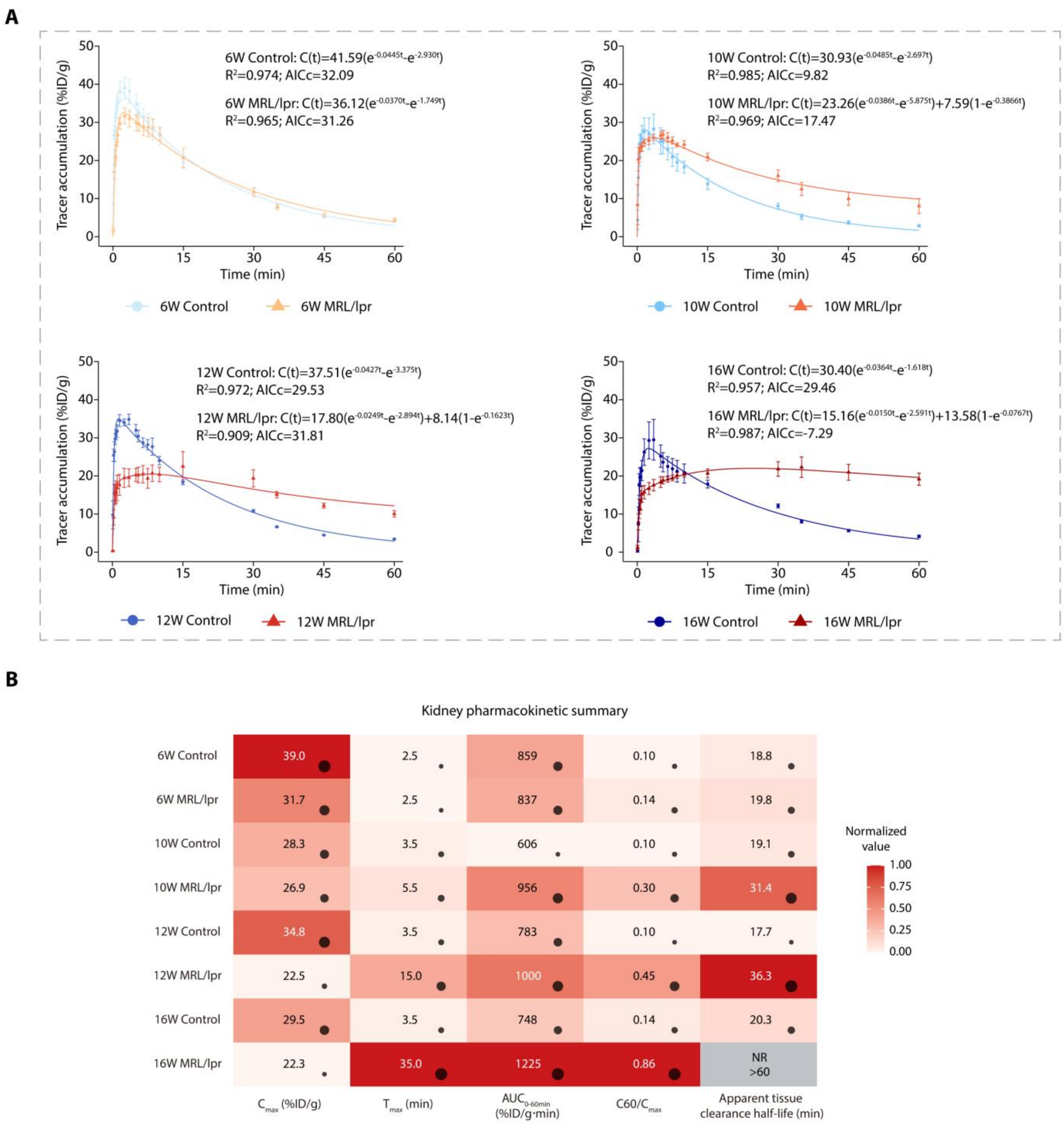

Fig. S4. Fitting of kidney uptake-clearance-retention model of [^{68}Ga]Ga-NOTA-ssDNA probe in MRL/lpr mice. (A) Curve fitting of the renal uptake-clearance-retention (UCR) model to probe kinetics in control and MRL/lpr mice at different

ages. (B) Overview of the pharmacokinetic parameters derived from the renal uptake-clearance-retention (UCR) model in control and MRL/lpr mice at different ages.

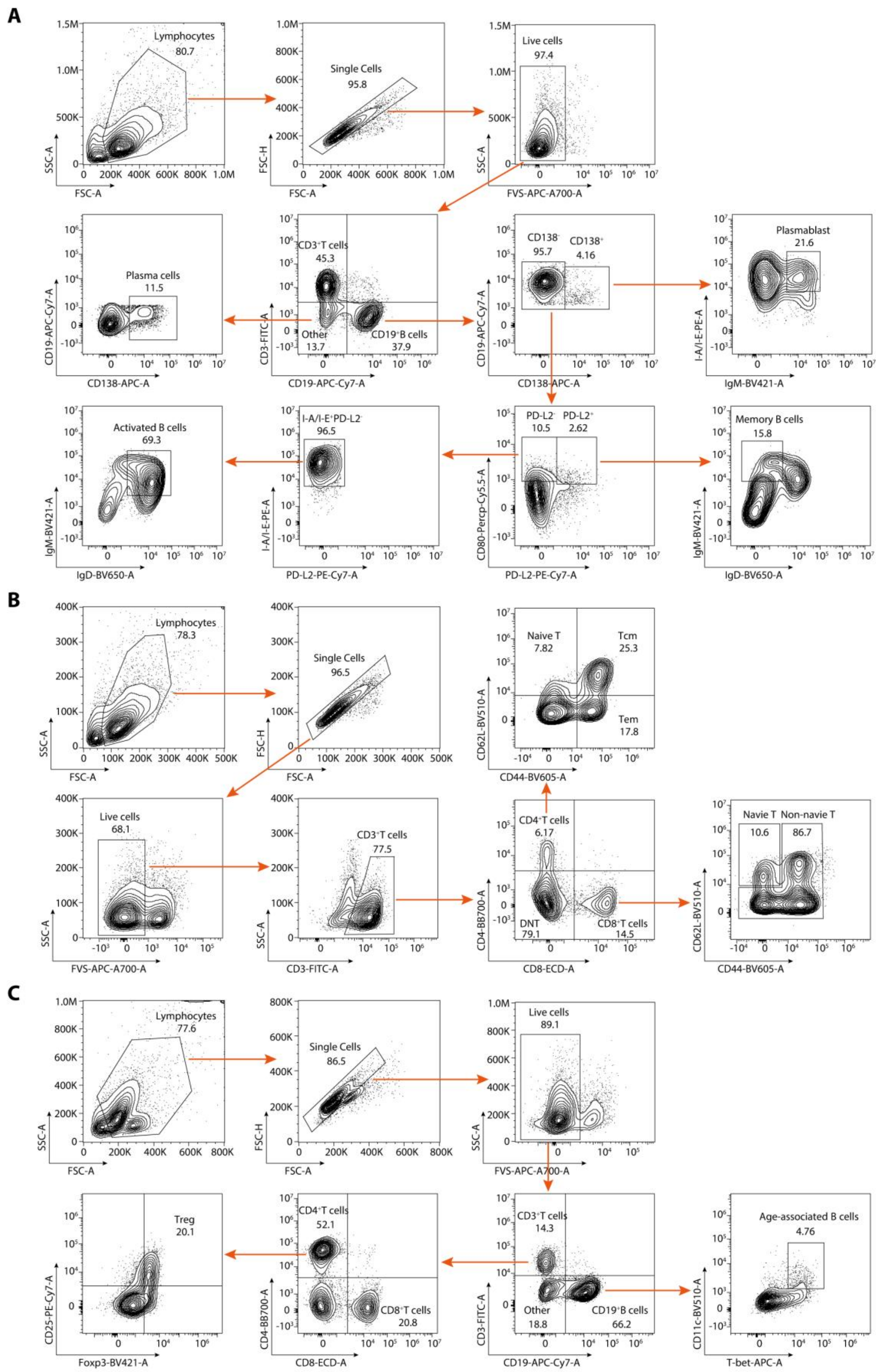

Fig. S5. Flow cytometric gating strategy for analyzing splenic immune cell subsets in MRL/lpr mice. (A) Gating strategy for B cell subset composition in the spleen of MRL/lpr mice. (B) Gating strategy for T cell subset composition in the spleen of

MRL/lpr mice. (C) Gating strategy for age-associated B cells (ABC) and regulatory T cells (Treg) in the spleen of MRL/lpr mice.

Table S1. Biodistribution of [^{68}Ga]Ga-NOTA-ssDNA in the Control group and MRL/lpr group at 16 weeks of age[a].

Tissue/organ	[^{68}Ga]Ga-NOTA-ssDNA in 16W Control	[^{68}Ga]Ga-NOTA-ssDNA in 16W MRL/lpr
Blood (%ID/g)	0.7970 ± 0.2978	3.5616 ± 1.0557
Brain (%ID/g)	0.0505 ± 0.0177	0.1164 ± 0.0294
Heart (%ID/g)	0.6480 ± 0.0988	1.1918 ± 0.2322
Lung (%ID/g)	0.5548 ± 0.1067	1.8741 ± 0.6840
Liver (%ID/g)	2.5132 ± 0.3872	4.6096 ± 1.7227
Spleen (%ID/g)	0.8852 ± 0.1212	1.2661 ± 0.3022
Kidney (%ID/g)	2.1287 ± 0.1939	8.7008 ± 0.5400
Stomach (%ID/g)	0.7185 ± 0.1061	0.7489 ± 0.0725
Small intestine (%ID/g)	0.5931 ± 0.0529	1.0442 ± 0.3105
Large intestine (%ID/g)	0.6497 ± 0.0746	1.0415 ± 0.0261
Muscle (%ID/g)	0.2941 ± 0.0391	0.6094 ± 0.0762
Bone (%ID/g)	0.3964 ± 0.0899	0.5369 ± 0.0286

[a] Data are presented as the mean ± SEM (n = 4).

Table S2. Primer sequences used for quantitative real-time PCR.

Gene	Forward primer (5'→3′)	Reverse primer (5′→3′)
mGapdh	GGTTGTCTCCTGCGACTTCA	TGGTCCAGGGTTTCTTACTCC
mTlr7	ATGTGGACACGGAAGAGACAA	GGTAAGGGTAAGATTGGTGGTG
mTlr9	ATGGTTCTCCGTCGAAGGACT	GAGGCTTCAGCTCACAGGG
mMyd88	TCATGTTCTCCATACCCTTGGT	AAACTGCGAGTGGGGTCAG
mIrf7	GAGACTGGCTATTGGGGGAG	GACCGAAATGCTTCCAGGG
mIfnar1	AGCCACGGAGAGTCAATGG	GCTCTGACACGAAACTGTGTTTT
mIrak4	CATACGCAACCTTAATGTGGGG	GGAACTGATTGTATCTGTCGTCG
mNfkb1	ATGGCAGACGATGATCCCTAC	TGTTGACAGTGGTATTTCTGGTG
mIfng	ATGAACGCTACACACTGCATC	CCATCCTTTTGCCAGTTCCTC
